# Transcriptomic Analysis Implicates the p53 Signaling Pathway in the Establishment of HIV-1 Latency in Central Memory CD4 T Cells in an In Vitro Model

**DOI:** 10.1371/journal.ppat.1006026

**Published:** 2016-11-29

**Authors:** Cory H. White, Bastiaan Moesker, Nadejda Beliakova-Bethell, Laura J. Martins, Celsa A. Spina, David M. Margolis, Douglas D. Richman, Vicente Planelles, Alberto Bosque, Christopher H. Woelk

**Affiliations:** 1 Graduate Program in Bioinformatics and Systems Biology, University of California San Diego, La Jolla, California, United States of America; 2 San Diego VA medical Center and Veterans Medical Research Foundation, San Diego, California, United States of America; 3 Clinical and Experimental Sciences, Faculty of Medicine, University of Southampton, Southampton, Hampshire, United Kingdom; 4 Department of Medicine, University of California San Diego, La Jolla, California, United States of America; 5 Division of Microbiology and Immunology, Department of Pathology, University of Utah School of Medicine, Salt Lake City, Utah, United States of America; 6 Department of Pathology, University of California San Diego, La Jolla, California, United States of America; 7 UNC HIV Cure Center, Department of Medicine, Microbiology and Immunology, University of North Carolina at Chapel Hill, Chapel Hill, North Carolina, United States of America; Emory University, UNITED STATES

## Abstract

The search for an HIV-1 cure has been greatly hindered by the presence of a viral reservoir that persists despite antiretroviral therapy (ART). Studies of HIV-1 latency *in vivo* are also complicated by the low proportion of latently infected cells in HIV-1 infected individuals. A number of models of HIV-1 latency have been developed to examine the signaling pathways and viral determinants of latency and reactivation. A primary cell model of HIV-1 latency, which incorporates the generation of primary central memory CD4 T cells (T_CM_), full-length virus infection (HIV_NL4-3_) and ART to suppress virus replication, was used to investigate the establishment of HIV latency using RNA-Seq. Initially, an investigation of host and viral gene expression in the resting and activated states of this model indicated that the resting condition was reflective of a latent state. Then, a comparison of the host transcriptome between the uninfected and latently infected conditions of this model identified 826 differentially expressed genes, many of which were related to p53 signaling. Inhibition of the transcriptional activity of p53 by pifithrin-α during HIV-1 infection reduced the ability of HIV-1 to be reactivated from its latent state by an unknown mechanism. In conclusion, this model may be used to screen latency reversing agents utilized in shock and kill approaches to cure HIV, to search for cellular markers of latency, and to understand the mechanisms by which HIV-1 establishes latency.

## Author Summary

The major hindrance to an HIV cure is the ability of the virus to persist in a latent state despite antiretroviral therapy. It is difficult to study this latent state in the HIV-infected patient because only a small proportion of cells in the body are affected and current technologies are not able to identify these cells. Therefore, models in the laboratory have been developed to study HIV latency. However, these models have not been adequately characterized with the latest genomic technologies. We have characterized our model of HIV latency using global gene expression analysis (i.e., RNA-Seq). Our model aims to reflect HIV latency in patients by using primary central memory CD4 T cells, wild type virus, and antiretroviral therapy. Our main finding was that signaling through the p53 protein characterized the latent state, and may be important in its establishment. This has implications for a better understanding of HIV latency which may lead to new therapies. In a broader context, we validated the latent state of our model of HIV latency, which can now be used with confidence to evaluate compounds used in strategies to cure HIV, search for markers of HIV latency, and further investigate the mechanisms leading to the establishment of latency.

## Introduction

A major obstacle to the eradication of HIV-1 is the persistence of the latent viral reservoir. While antiretroviral therapy (ART) has been extremely effective at suppressing viral replication, it has not eradicated this reservoir [1]. Upon the removal of ART, HIV-1 emerges from the latent state and replicates to levels akin to an acute infection that leads to disease progression [2,3]. The low frequency of latently infected cells within the HIV-1 patient (between 1 and 60 in 10^6^ resting CD4 T cells) complicates the study of this viral reservoir *in vivo* [4,5,6]. This has prompted the development of models of HIV-1 latency based on chronically infected cell lines and primary human CD4 T cells [7]. To obtain an accurate representation of HIV-1 latency *in vivo*, it is essential to fully characterize these different models.

Transcriptome profiling by microarrays or RNA-Seq allows the simultaneous evaluation of transcriptional activity of the entire genome within a sample, thus providing a comprehensive analysis of the biological condition of the cell population at a given time. These technologies are becoming important for the study of HIV-1 latency, particularly in the search for biomarkers of HIV-1 latency [8,9] and for evaluating the effects of latency reversing agents [10,11,12,13]. Krishnan and Zeichner [14], utilized cDNA spotted microarrays to compare gene expression in cell lines chronically infected with HIV-1 (i.e., ACH-2, J1.1, U1) and their parental uninfected lines to identify 32 genes that were consistently differentially regulated. The laboratory of Fabio Romerio used Agilent microarrays to profile latently infected and uninfected conditions from four donors using their primary CD4 T cell latency model, and a gene encoding a surface receptor, *CD2*, was identified to be enriched in latently infected cells [9].

RNA-Seq is the current state-of-the-art technology with respect to transcriptomics and is thought to exhibit greater specificity and dynamic range than microarrays [15]. The first RNA-Seq study of a primary CD4 T cell model of latency incorporated a GFP expressing virus [16]. When samples from a single donor were profiled over time, a large number of genes were identified as dysregulated during the latent phase (N = 227) and were associated with chemokine receptors, cytokine signaling, and general immune responses. We previously used RNA-Seq to profile latently infected and uninfected samples from 4 donors [17] from the first iteration of a cultured primary central memory CD4 T cell (T_CM_) model [18,19]. This study demonstrated that the defective vectors used to seed the latent reservoir were recombining to reconstitute actively replicating HIV-1. This observation led to the revision of the cultured T_CM_ model of HIV-1 latency by incorporating wild type virus (HIV_NL4-3_) and ART to suppress virus replication [20].

The purpose of the present RNA-Seq study was primarily to identify mechanisms involved in the establishment of HIV-1 latency but also to characterize this modified cultured T_CM_ model of latency [20] to confirm that the model reflects a latent state. Comparison of latently infected to uninfected cells identified differential expression of genes in the p53 signaling pathway. Treatment with pifithrin-α, an inhibitor of the transcriptional activity of p53 [21], reduced the ability of latently infected cells to be reactivated in the cultured T_CM_ model. The main finding from these results suggests a direct effect of p53 on the establishment and ability to reactivate the latent reservoir.

## Results

### Generation of latently infected cultured T_CM_ cells

**Fig 1:**
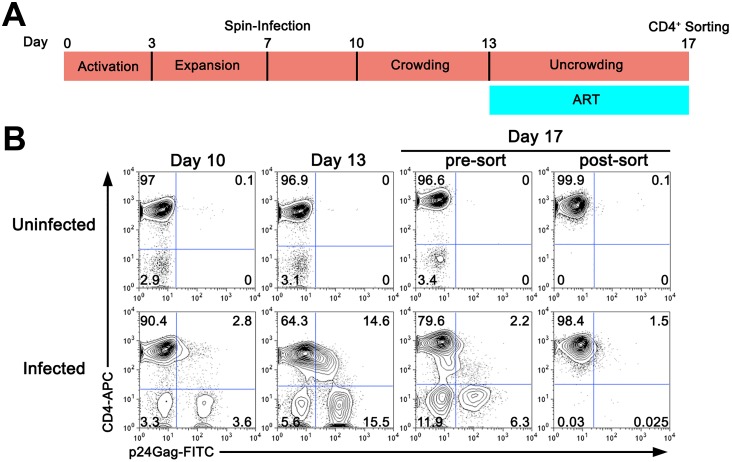
Generation of latently infected cultured T_CM_. (A) Time-line of experimental procedure. A full description of the methodology is provided in Materials and Methods. (B) Analysis of infection in a representative donor. At the time points indicated, CD4 expression and HIV-1 p24 Gag expression was measured by flow cytometry as indicated in Materials and Methods. X-axis indicates HIV-1 p24 Gag staining and Y-axis CD4 staining. At day 17, cells were sorted based on surface CD4 expression. Staining of the cells pre- and post-sorting is indicated. Numbers in dot-plots represent percentages.

To dissect the viral and cellular status of the cultured T_CM_ model of latency [20], gene expression profiles were generated by RNA-Seq for a total of 16 samples from 4 different donors representing the following 4 conditions: uninfected (UI), latently infected (LI), uninfected and activated (UIA), and latently infected and activated (LIA). Briefly, naïve CD4 T cells from four HIV-1 negative individuals were isolated and activated with αCD3/αCD28 beads for Three days in conditions that generate central memory CD4 T cells (Fig 1A) [18]. After activation, cells were allowed to expand in the presence of IL-2 and infected by spinoculation at day 7 with HIV-1_NL4-3_ at a low MOI that rendered 3–7% of cells infected at day 10 (Fig 1B, day 10). Cells were then seeded for an additional three-day period in a 96 well (round bottom) plate to increase the efficiency of virus transmission (Fig 1B, day 13). At that time point, cells were diluted and cultured for 4 extra days in the presence of IL-2 and ART (Raltegravir plus Nelfinavir). At day 17, CD4 positive T cells were isolated using magnetic bead sorting. This strategy was chosen because productively infected cells downregulate CD4 expression on the cell surface due to the expression of the accessory genes Nef and Vpu (Fig 1B, day 17) [22,23]. This procedure largely eliminates productively infected (p24 positive) cells, as well as CD4 negative cells present in the culture. Therefore, cells in the UI and LI conditions were CD4 positive cells that were not expressing detectable levels of viral antigens. UI and LI cells were further activated with αCD3/αCD28 beads in the presence of ART for 48 hours to generate cells for the UIA and LIA conditions.

### Cultured T_CM_ cells have a quiescent phenotype

**Fig 2:**
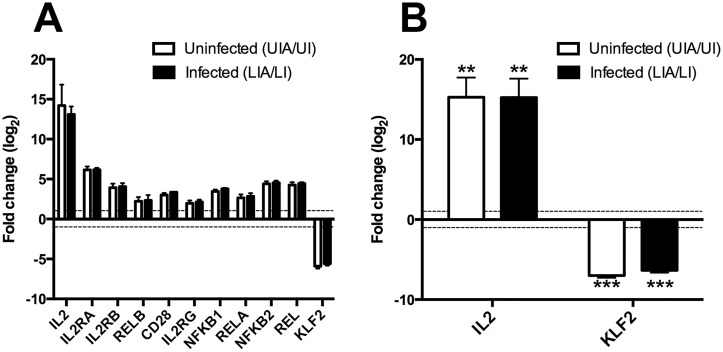
Markers of CD4 T cell activation are modulated following αCD3/αCD28 stimulation. The fold change of a panel of host gene markers of CD4 T cell activation was assessed pre- and post-stimulation with αCD3/αCD28 beads by (A) RNA-Seq and (B) RT-qPCR. Fold changes were calculated for both uninfected (white bars) and latently infected (black bars) cells. Fold changes for all genes assessed by RNA-Seq were significant (FDR corrected *p*-value <0.05). Fold changes for *IL2* and *KLF2* assessed by RT-qPCR were significant in a paired *t*-test at the following levels: **p<0.01 ***p<0.001. Fold change is plotted on the log_2_ scale with error bars representing standard deviation. The horizontal dotted line in each figure indicates a log_2_ fold change cut off of 1 (i.e., actual fold change cut off of 2). Abbreviations are as follows: *UI*, uninfected; *UIA*, uninfected activated; *LI*, latently infected; *LIA*, latently infected activated.

Initially, the resting and activated conditions were compared (i.e., UI vs. UIA and LI vs. LIA) to validate the phenotype of the resting cells. When comparing RNA content between these conditions, transcriptional amplification had occurred (S1 Fig). Traditional normalization procedures for transcriptomic data do not account for transcriptional amplification [24,25]. Therefore, Biological Scaling Normalization (BSN) using ERCC spike-in control RNAs was used to normalize RNA-Seq data to allow comparison of resting and activated conditions [26] (See Materials and Methods). A number of gene expression markers of CD4 T cell activation were modulated following activation in both the LIA and UIA conditions (Fig 2A). Notably, *IL2* and components of its receptor (*IL2RA*, *IL2RB*, *IL2RG*) were upregulated upon activation, as were members of the NFκB complex (*NFKB1*, *NFKB2*, *REL*, *RELA*, *RELB*), and *CD28* itself. The *KLF2* gene, which is highly upregulated in quiescent CD4 T cell lymphocytes, but repressed during activation [27,28], was significantly downregulated as expected. The modulation of *IL2* and *KLF2* upon T cell activation was further confirmed by RT-qPCR (Fig 2B). In summary, resting cultured T_CM_ cells have the phenotypic characteristics of a quiescent T cell and stimulation with αCD3/αCD28 beads modulates known markers of CD4 T cell activation.

### Activation of HIV-1 from a latent state

**Fig 3:**
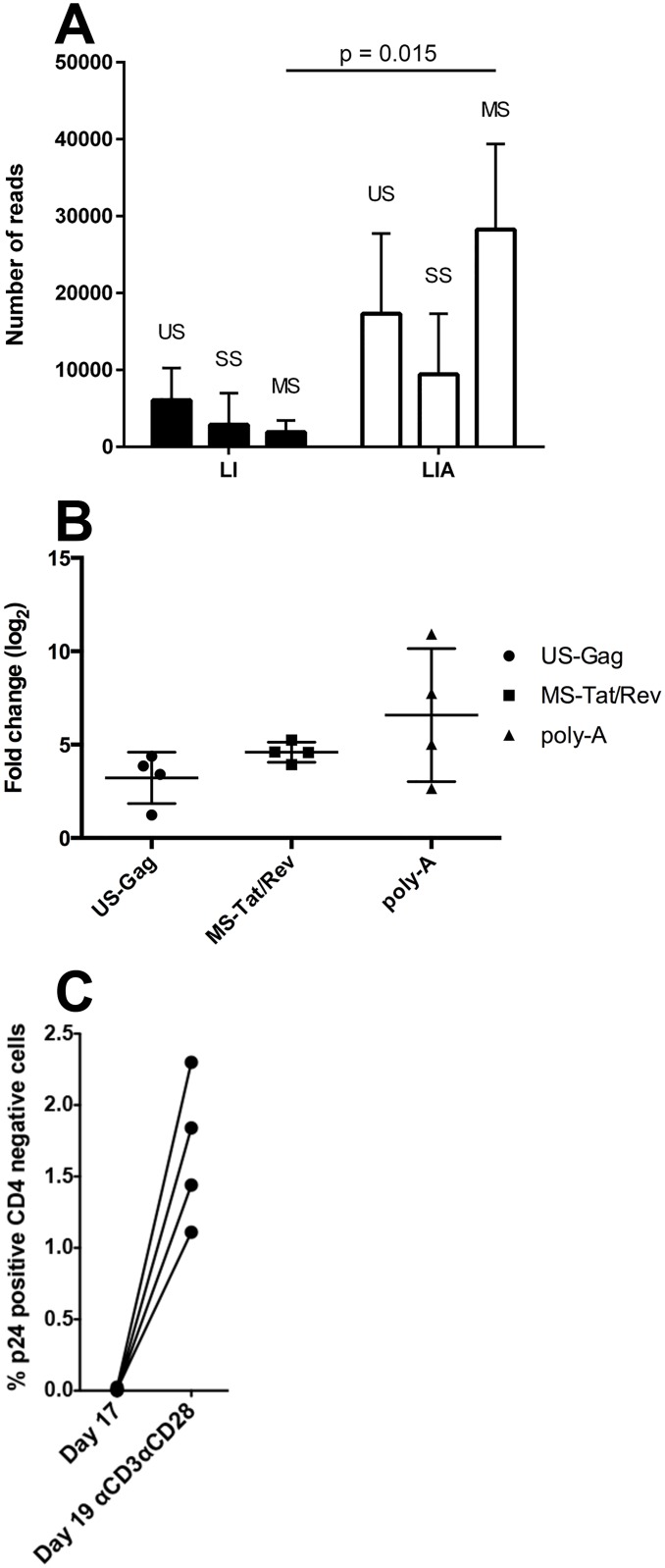
HIV-1 splicing variants are upregulated following αCD3/αCD28 stimulation. (A) The abundance of HIV-1 unspliced (US), singly spliced (SS), and multiply spliced (MS) transcripts in the LI and LIA conditions was estimated by mapping reads over the two major splice sites D1 and D4 (see Materials and Methods for details). No more than 4 reads were identified to map to the HIV-1 genome in samples from the uninfected conditions (UI and UIA), which may reflect expression from endogenous retroviral elements in the human genome. The significance of increases in each class of HIV reads upon activation was assessed using a one-tailed paired t-test but only an increase in MS reads was identified as significant (*p* = 0.015). (B) Fold changes upon activation of US RNA (US-Gag), MS RNA (MS-Tat/Rev) and polyadenylated RNA were determined by RT-qPCR. Significance of fold changes was confirmed using a one-tailed paired t-test and all RT-qPCR measurements were identified as significant at the *p*<0.001 level. Fold change is plotted on the log_2_ scale with error bars representing standard deviation. (C) Surface CD4 and intracellular HIV-1 p24 Gag expression were measured by flow cytometry as indicated in Materials and Methods after isolation and after reactivation with αCD3/αCD28 beads for 48 hours.

Next, the effect of antigen stimulation mimicked by αCD3/αCD28 beads on HIV-1 transcription was evaluated. HIV-1 transcription in the resting (LI) and activated (LIA) states was compared after BSN. Treatment with αCD3/αCD28 beads induced global upregulation of total HIV-1 reads from the resting to the activated conditions (average 6.6 fold change, s.d. ±3.6, *t*-test *p*-value = 0.04) with an increase in all major splicing groups: unspliced (US), singly spliced (SS), and a significant increase (*p* = 0.015) in multiply spliced (MS) (Fig 3A). A significant increase of US, MS and total polyadenylated HIV-1 transcripts upon activation was confirmed by RT-qPCR (Fig 3B) with a concomitant increase in HIV-1 p24 protein (Fig 3C). The fold change increase in polyadenylated transcripts appears much more variable than US or MS transcripts (Fig 3B). It is unclear what is driving this variation but measurements of polyadenylated transcripts reflect fully elongated and correctly processed HIV-1 mRNA, which relies on the host transcriptional machinery. It is possible that the efficiency of polyadenylation varies across donors, whereas US and MS are less variable because they measure HIV transcripts, whether polyadenylated or not. In support of this, single nucleotide polymorphisms that vary between donors and effect post-transcriptional processing and subsequent gene expression have previously been identified [29]. In summary, examination of HIV-1 US, MS and polyadenylated transcripts further confirmed that the LIA condition of the cultured T_CM_ model reflected activation of transcription from an HIV-1 latent state (LI).

### Perturbation of host gene expression in latently infected cells

**Fig 4:**
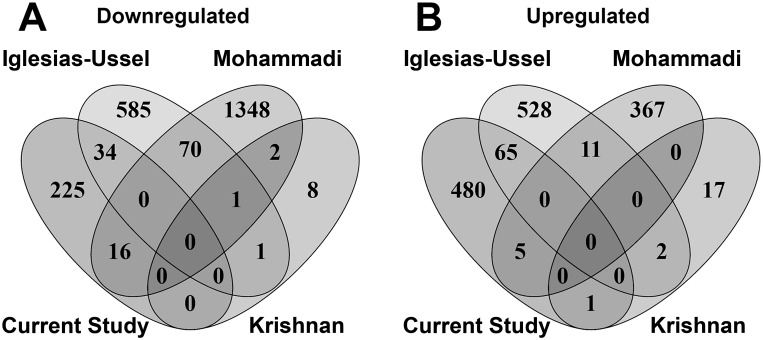
Comparison of genes dysregulated in latency across four *in vitro* models of HIV-1 latency. The comparison between DEGs across four models of HIV-1 latency was performed by constructing a Venn Diagram with tool Venny 2.1.0. All differentially expressed genes were separated into (A) downregulated and (B) upregulated genes and then compared across the following models: the current study (RNA-Seq results from the cultured T_CM_ model of HIV-1 latency [20] for 4 donors), Iglesias-Ussel et al. [9] (microarray results generated from a primary CD4 T cell model for 4 donors), Mohammadi [16] (RNA-Seq results from a primary CD4 T cell model for a single donor), and Krishnan and Zeichner [14] (microarray results generated by overlapping DEGs from three HIV-1 infected cell line models).

The UI and LI samples were compared to identify 826 differentially expressed genes (DEGs, S1 Table), 275 of which were downregulated and 551 upregulated (false discovery rate [FDR] corrected *p*-value < 0.05). The top 100 DEGs presented in the heatmap (S2 Fig) demonstrated the consistency of gene expression across donors. Although a large signal of differentially expressed genes was identified between the UI and LI conditions, it should be noted that this signal may be confounded by low proportions of latently infected cells and bystander effects. Specifically, a small proportion of latently infected cells in a background of uninfected cells is being compared to a population of 100% uninfected cells. The possible impact of this potential limitation is fully expanded upon in the Discussion section of this manuscript. The DEGs identified in the analysis were compared to other primary CD4 T cell [9,16] and cell line models of latency [14]. Although no up- or downregulated DEGs were identified in common across all models, (Fig 4A & 4B), greater overlap was identified when comparing primary cell models in a pairwise fashion. In particular, there were 65 genes upregulated during latency in common between our cultured T_CM_ model [20] and the model used by Iglesias-Ussel et al. [9] (S2 Table). Unfortunately, the upregulation of *CD2* during latent infection previously identified by Iglesias-Ussel et al. [9] was not confirmed in our T_CM_ model. One explanation to this result is that in the model used by Iglesias-Ussel et al. [9], the cells isolated to perform the analysis were expressing intracellular p24. Our cultured T_CM_ model of HIV-1 latency largely eliminates such cells.

### Functional analysis of genes perturbed during latency

**Fig 5:**
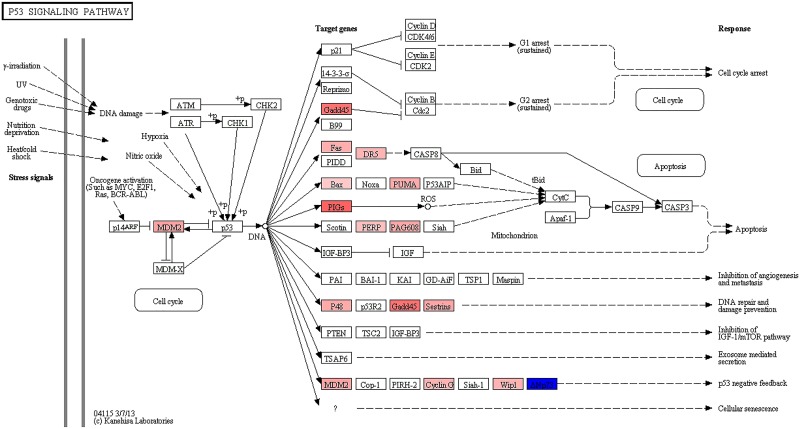
Differentially expressed genes from the p53 signaling pathway during HIV-1 latency. Gene expression changes overlaid upon the KEGG *p53 signaling pathway* for all differentially expressed genes with an FDR corrected p-value <0.05. The shade of red indicates the degree of upregulation while the shade of blue indicates the degree of downregulation.

In order to develop a better understanding of genes perturbed during latency in the cultured T_CM_ model, the 826 genes differentially expressed between the LI and UI conditions were subjected to pathway and protein interaction (PIN) analysis. The only Kyoto Encyclopedia of Genes and Genomes (KEGG) pathway that attained significance for over-representation of DEGs was the "*p53 signaling pathway*" (FDR corrected *p*-value = 5.5E-06). The results of differential gene expression were overlaid on the *p53 signaling pathway* and revealed that multiple threads related to *apoptosis* and *DNA repair and damage prevention* were upregulated in latently infected cells [30] (Fig 5). Of note, a number of p53 related genes (*ACTA2*, *BBC3*, *DDB2*, *DRAM1*, *FDXR*, *GADD45A*, and *TNFRSF10B*) were present in the 65 upregulated genes (S2 Table) in common between our study and that of Iglesias-Ussel et al. [9].

**Fig 6:**
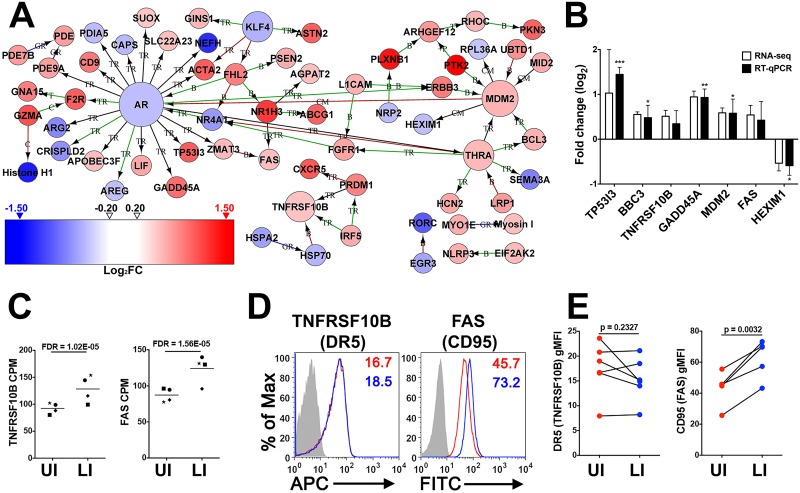
Functional analysis of genes modulated in HIV-1 latency. (A) Protein interaction network (PIN) generated using differentially expressed genes (N = 826) after a log_2_FC > 0.5 filter was applied (N = 64). Any single nodes not attached to a main hub were removed for clarity. Abbreviations for the type of interaction are as follows: *B*–Binding, *C*–Cleavage, *CM*–Covalent Modification, *GR*–Group Relation, *+P*–Phosphorylation, *TR*–Transcription Regulation, *T*–Transformation. Edge coloring of Green indicates activation, Red indicates inhibition, Black indicates unspecified, while Blue is utilized for a group relation. Arrows indicate the direction of interactions. The color gradient of log_2_FC for (A) is included with red indicating upregulation and blue indicating downregulation. (B) RT-qPCR validation of a panel of p53 related genes modulated in HIV-1 latency. Fold change is plotted on the log_2_ scale with error bars representing standard deviation. Significance for RT-qPCR results was determined with a paired *t*-test (*p<0.05, **p<0.01, ***p<0.001) and all RNA-Seq results were significant (EdgeR’s FDR corrected *p*-value < 0.05). (C) Dot plots of gene expression data (i.e., counts per million) from RNA-Seq results for *TNFRSF10B* (DR5) and *FAS* (CD95). Abbreviations: *UI*, uninfected; *LI*, latently infected. (D) Histograms of DR5 (*TNFRSF10B*) and CD95 (*FAS*) protein surface expression from a representative donor. Red line indicates UI and blue line indicates LI. Geometric mean fluorescence intensity (gMFI) is indicated at the right top corner of the histogram and the grey histogram represents unstained control. (E) Surface expression was measured by FACS for DR5 (6 donors) or CD95 (5 donors). Significance was determined with a paired *t*-test (*p*-values provided).

A PIN constructed using genes differentially expressed during latency complemented KEGG pathway analysis by confirming the importance of genes related to p53 activity (Fig 6A and Table 1). This PIN contained two major hubs (*AR* and *MDM2*) and one minor hub (*TNFRSF10B a*.*k*.*a*. *DR5* or *TRAIL-R2*), which are all related to p53 activity. For example, *MDM2* facilitates negative feedback to the p53 signaling pathway by degrading p53 and the upregulation of this gene in the LI condition may be indicative of prior p53 activity [31]. *AR* is negatively regulated by p53 signaling [32], and correspondingly, is downregulated in the LI condition. *TNFRSF10B*, a gene involved in programmed cell death, is upregulated and known to be induced by p53 in response to DNA damaging agents [33]. Therefore, the PIN extended KEGG pathway analysis by identifying p53 related hub genes (e.g. *AR*) and their targets that were not curated into the KEGG *p53 signaling pathway*.

**Table 1:** Genes responsive to p53 activity.

Symbol	Full Gene Name	FC	FDR	Citation
***MDM2***	**MDM2 proto-oncogene, E3 Ubiquitin Protein Ligase**	**1.503**	**8.688E-06**	**Uniprot 2015** [31]
*BCL3*	B-Cell CLL/Lymphoma 3	1.517	4.338E-02	Kashatus et al., 2006 [34]
*HEXIM1*	Hexamethylene Bis-Acetamide Inducible 1	-1.449	3.251E-05	Lew et al., 2012 [35]
*PTK2*	Protein Tyrosine Kinase 2	3.060	1.748E-07	Uniprot 2015 [31]
*RHOC*	Ras Homolog Family Member C	1.494	2.900E-04	Croft et al., 2011 [36]
*UBTD1*	Ubiquitin Domain Containing 1	1.698	1.893E-03	Zhang et al., 2015 [37]
*NRP2*	Neuropilin 2	-1.464	1.234E-03	Arakawa et al., 2005 [38]
***AR***	**Androgen Receptor**	**-1.453**	**1.796E-02**	**Alimirah et al., 2007** [32]
*ACTA2*	Actin, Alpha 2, Smooth Muscle, Aorta	2.033	1.946E-08	Comer et al., 1998 [39]
*AREG*	Amphiregulin	-1.439	4.697E-02	Taira et al., 2014 [40]
*FAS*	Fas Cell Surface Death Receptor	1.455	1.534E-05	Kim et al., 1999 [41]
*FHL2*	Four and a Half LIM Domains 2	1.803	3.223E-03	Xu et al., 2014 [42]
*GADD45A*	Growth Arrest and DNA-Damage-Inducible, Alpha	1.924	1.054E-10	Jin et al., 2003 [43]
*NR4A1*	Nuclear Receptor Subfamily 4, Group A, Member 1	-1.656	3.151E-02	Zhao et al., 2005 [44]
*TP53I3*	Tumor Protein p53 Inducible Protein 3	2.039	7.704E-04	Maglott et al., 2005 [45]
***TNFRSF10B***	**Tumor Necrosis Factor Receptor Superfamily, Member 10b**	**1.425**	**1.007E-05**	**Takimoto and El-Deiry 2000** [33]
*CXCR5*	Chemokine (C-X-C Motif) Receptor 5	2.073	4.235E-04	Mitkin et al., 2015 [46]
*IRF5*	Interferon Regulatory Factor 5	1.548	9.405E-03	Mori et al., 2002 [47]
*PRDM1*	PR Domain Containing 1, with ZNF Domain	1.785	1.290E-06	Yan et al., 2007 [48]

This table shows genes involved in p53 activity that are dysregulated during latency identified in the protein interaction network (PIN, Fig 6A). Highlighted in bold are the central hub genes from the PIN. Non-hub genes are grouped based upon their direct or indirect connection to a primary hub gene. Fold change (FC) was calculated as LI/UI and the corresponding FDR-corrected *p*-value presented.

Several genes (*BBC3*, *FAS*, *GADD45A*, *HEXIM1*, *MDM2*, *TNFRSF10B* and *TP53I3*) selected from the *p53 signaling pathway* and the PIN were subjected to RT-qPCR analysis (Fig 6B). The upregulation of transcripts of *BBC3*, *GADD45A*, *MDM2*, *TP53I3*, and downregulation of *HEXIM1* during latent infection was confirmed as significant by RT-qPCR. The direction of fold change was validated by RT-qPCR for the cell surface markers *FAS* and *TNFRSF10B* (Fig 6B), which were previously noted as significantly upregulated in the RNA-Seq data (Fig 6C). The cell surface expression of these markers was interrogated in an independent donor set by flow cytometry (Fig 6D) but only *FAS* (CD95) was confirmed as significantly upregulated on the surface of T cells (fold change of 1.47 ± 0.20, Fig 6E). In summary, functional analysis of genes perturbed during latency using pathway and PIN analyses identified dysregulation of genes associated with p53 activity.

### Inhibition of p53 signaling perturbs the latent reservoir *in vitro*

**Fig 7:**
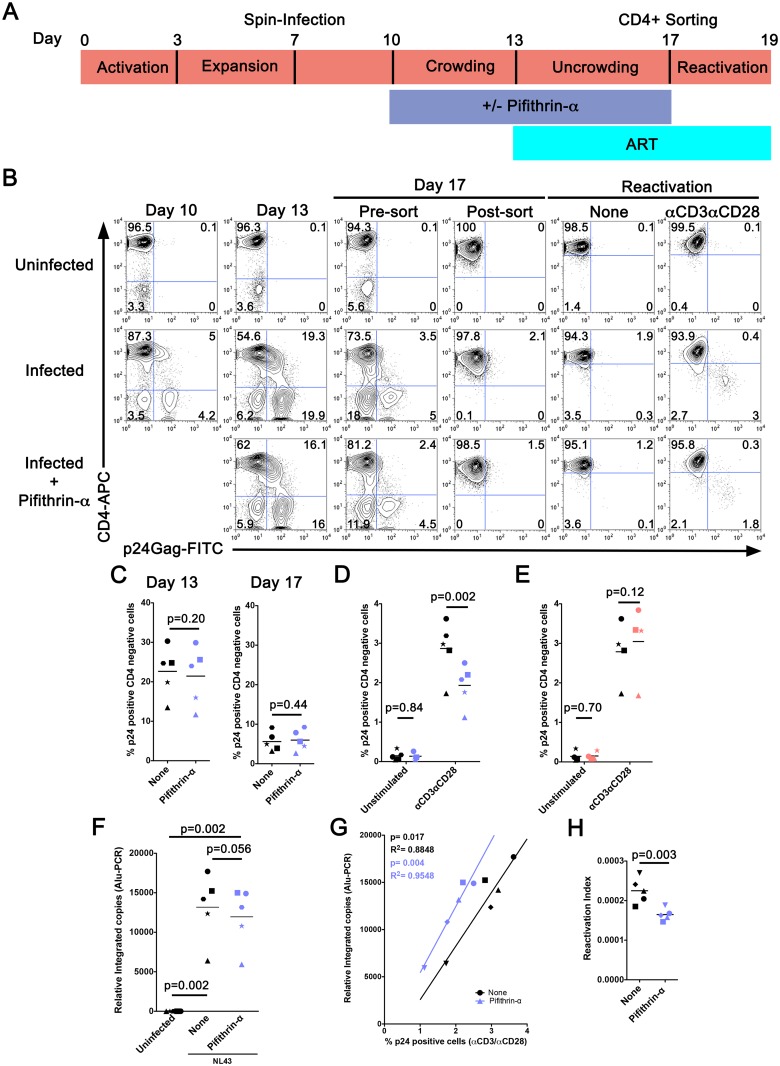
Effect of pifithrin-α upon establishment of latency. (A) Time-line of experimental procedures for pifithrin-α treatment of the latently infected (LI) condition. For the majority of experiments pifithrin-α was maintained in culture from day 10 to 17. (B) CD4 and p24 Gag staining from a representative donor throughout the experiment. Numbers in dot-plots represent percentages. (C) p24 Gag positive CD4 negative cells from 5 independent donors at day 13 and day 17 in cells treated (purple symbols) or untreated (black symbols) with pifithrin-α. (D) p24 Gag positive CD4 negative cells at day 19 following reactivation of LI cells with αCD3/αCD28 beads at day 17 in cells previously treated (purple symbols) or untreated (black symbols) with pifithrin-α (day 10 to 17). As a negative control, p24 Gag positive CD4 negative cells were also assessed in unstimulated cells treated with pifithrin-α in a similar manner. (E) In a deviation from the time-line presented in (A), pifithrin-α was added only during the reactivation period from day 17 to 19. p24 positive CD4 negative cells were then determined at day 19 following reactivation of LI cells that were treated (salmon symbols) or untreated (black symbols) with pifithrin-α. As a negative control, p24 Gag positive CD4 negative cells were also assessed in unstimulated cells treated with pifithrin-α in a similar manner. (F) HIV-1 integration analysis in LI cells at day 17 using Alu-PCR with 250 ng of genomic DNA. As a negative control, HIV-1 integration was also assessed in uninfected cells. (G) Correlation of integrated HIV-1 with the percentage of p24 Gag positive CD4 negative cells after reactivation with αCD3/αCD28 beads. Correlation was determined using the Pearson correlation coefficient. Black symbols represent untreated samples and purple symbols represent cells treated with pifithrin-α from day 10 to 17. (H) Reactivation index calculated as the percentage of cells expressing p24 (day 19) after αCD3/αCD28 reactivation divided by the number of integrated copies before reactivation (day 17). Throughout this figure each donor is represented with a different symbol and significance was determined with a paired *t*-test (*p*-values provided).

Pifithrin-α, an inhibitor of p53 transcriptional activity [21], was added to CD4 T cells from five additional donors after the initial infection at day 10 and again at day 13 in the cultured T_CM_ model to investigate the possible role of p53 signaling in HIV-1 latency (Fig 7A). Inhibition of p53 had no effect on HIV-1 replication since no difference was observed in the levels of p24 between treated and untreated samples at days 13 and 17 (Fig 7B and 7C). However, inhibition of p53 transcriptional activity using pifithrin-α resulted in a reduction of the number of cells producing p24 (average reduction of 32%, s.d. ±8%) after reactivation with αCD3/αCD28 beads (Fig 7B and 7D). Interestingly, when pifithrin-α was added only during the reactivation step (day 17), there was no effect on viral reactivation (Fig 7E). These results suggest that inhibition of p53 during the active replication phase of HIV-1 may have an effect on the establishment or maintenance of HIV-1 latency. To test this hypothesis, integrated HIV-1 was analyzed by Alu-PCR in LI cells treated or not treated with pifithrin-α. There was a trend towards the reduction of integrated HIV-1 in pifithrin-α treated samples (Fig 7F, p = 0.056). However, the reduction on integration (average reduction of 9%, s.d. ±5%) does not fully explain the reduction in p24 observed after reactivation with αCD3/αCD28 beads (Fig 7D, average reduction of 32%, s.d. ±8%). Therefore, it is possible that in addition to reducing the number of integrated copies of HIV that pifithrin-α may be further inactivating the integrated provirus, albeit through an unknown mechanism. To support this, integrated copies of the provirus were correlated with the percentage of p24 producing cells following reactivation independently for pifithrin-α treated and untreated cells (Fig 7G). The correlation lines in this plot are parallel and shifted to the left for pifithrin-α treatment suggesting that the integrated virus in cells treated with pifithrin-α may be less prone to reactivation with αCD3/αCD28 beads. To compare both populations, we calculated the reactivation index measured as the percentage of cells expressing p24 after αCD3/αCD28 reactivation divided by the number of integrated copies before reactivation. This index compares the ability of an integrated copy to be reactivated by αCD3/αCD28 beads. Interestingly, cells that were treated with pifithrin-α have a lower ability to reactivate latent HIV-1 (Fig 7H). In summary, these studies confirmed that p53 signaling may play an important role in the establishment and maintenance of HIV-1 latency in this cultured T_CM_ model.

## Discussion

In this study, RNA-Seq was utilized to characterize a cultured T_CM_ model of HIV-1 latency [20], which incorporates a replication-competent virus (HIV-1_NL4-3_) and ART to suppress HIV-1 replication. RNA-Seq analysis demonstrated that the resting condition in this model (LI) reflects a quiescent and a latent state when compared to the activated state (LIA) both at the level of host and virus transcription. Notably, an increase in HIV-1 transcription was observed in all donors after activation (Fig 3A), indicative of a departure from a latent state [49].

The result of subtracting MS from US reads in the LI condition (mean difference 4,134, s.d. ±5448) was significantly different (*p* = 0.03, paired t-test) than this subtraction in the LIA condition (mean difference -10,928, s.d. ±3,408). This demonstrates a significant shift from US to MS reads upon activation suggesting an increase in early HIV-1 transcripts (Vpr, Tat, Rev, and Nef) after activation. The greater numbers of US versus MS reads in the LI condition of the T_CM_ model is supported by previous reports that consistently detect a greater signal for US over MS transcripts in the resting CD4 T cells isolated from HIV-1 infected patients on ART [50,51]. The increase in HIV-1 transcription was not solely due to abortive transcripts since polyadenylated viral transcripts, which reflect fully elongated and correctly processed HIV-1 mRNA, were significantly upregulated after activation.

By comparing expression profiles from UI to those from LI cells, a total of 826 differentially expressed genes were identified (S2 Table). While there was only minimal overlap in genes dysregulated during latency when comparing this cultured T_CM_ model to published data from three additional models of HIV-1 latency (Fig 4A & 4B), there appeared to be greater overlap between primary CD4 T cell models compared to models based on cell lines (S2 Table). Several reasons may account for these differences. First, this overlap might have been greater if the same transcriptomic technologies had been utilized, e.g. Iglesias-Ussel et al. [9] used the Agilent-012391 Whole Human Genome Oligo Microarray (G4112A) to profile gene expression compared to RNA-Seq in this study. Second, the RNA-Seq study of Mohammadi et al. [16] analyzed samples from only a single donor. Greater overlap between primary HIV-1 latency models may occur when better powered transcriptomic studies (i.e., multiple donors) are performed using the state-of-the-art technology (i.e., RNA-Seq).

Functional analysis of DEGs identified between the LI and UI conditions clearly implicated the transcriptional activation of genes by p53 (Figs 5 and 6, and Table 1). For example, the genes *ACTA2*, *BBC3 (a*.*k*.*a*. *Puma)*, *DDB2*, *DRAM1*, *FAS*, *FDXR*, *GADD45A*, *PRDM1*, *RHOC*, *TNFRSF10B*, and *TP53I3* are known to be induced by p53 [33,36,39,43,45,48,52,53,54], and were upregulated in the LI condition, which is in agreement with previous studies showing the activation of the p53 pathway mediated by HIV-1 through type I IFN signaling [55,56,57,58]. Of these genes, *FAS* (CD95) was further confirmed at the protein level (Fig 6E). In addition to upregulated p53 related genes, activation of p53 by genotoxic stress has been shown to result in downregulation of *AR* expression [32], also downregulated in this T_CM_ model (Fig 6A). The p53 protein itself is highly regulated and its activity must be tightly controlled to allow normal cellular functioning [59]. To maintain homeostasis, p53 will not only activate genes that enhance and stabilize its activity but also genes that repress its activity through negative feedback loops. Further evidence of prior p53 signaling activity was demonstrated by the upregulation of genes which act to degrade p53 following activation (Table 1). For instance, the protein product of *MDM2* mediates ubiquitination and breakdown of p53 resulting in the inhibition of p53-mediated apoptosis [31], and was upregulated in the LI condition (fold change = 1.503). Several other genes also involved in the degradation or inhibition of p53 [31,34,35,44,60,61], were upregulated in the LI condition: *BCL3*, *CCNG1*, *HEXIM1*, *LIF*, *NR4A1*, and *PTK2*. In summary, the latently infected (LI) condition in the T_CM_ model of HIV-1 latency exhibited evidence of prior p53 activation and subsequent negative feedback of this signaling pathway.

The identification of p53 related genes modulated during HIV-1 infection led to the hypothesis that this pathway may be important for the establishment of latency. Experiments with the p53 inhibitor pifithrin-α demonstrated that inhibition of this pathway did not affect viral replication or cell death, but did limit the number of cells that could be reactivated from latency (S3 Fig, Fig 7C, 7D and 7E). A number of explanations can account for these results. First, Alu-PCR analysis suggested that p53 may be required for successful integration of HIV-1 (Fig 7F). The p53 protein is not only involved in apoptosis and cell cycle arrest, but also in the activation of DNA repair mechanisms [62]. A number of p53-responsive genes identified in our study (*BBC3*, *DDB2*, *GADD45A*, *FDXR*, *PCNA* and *XPC*) are related to radiation-induced DNA damage [63] and point towards involvement of the nucleotide excision repair (NER) pathway, a process that recognizes and removes helix-distorting DNA lesions from the genome [64,65]. For example, GADD45A binds to UV-damaged DNA where it is believed to modify DNA accessibility within chromatin [66]. Although prior studies have primarily implicated other DNA repair pathways such as non-homologous end joining and base excision repair in HIV-1 DNA integration [67,68], the results of the present study suggest the p53-responsive genes that are components of the NER pathway may also play a role in the establishment of latency. Interestingly, a previous siRNA screen to characterize DNA repair factors involved in HIV-1 integration demonstrated that siRNA knockdown of *DDB2*, a component of the NER pathway, resulted in a large reduction (71.3%) of HIV-1 integration [67].

A second hypothesis to explain the effects observed by pifithrin-α could be the silencing of the HIV-1 provirus. It should be noted, that despite approaching significance and in the same direction for each donor, the reduction in HIV integration by pifithrin-α is modest (Fig 7F), and does not correspond entirely to the magnitude of the difference in p24 following reactivation (Fig 7D). Therefore, in addition to reducing the establishment of latency, pifithrin-α may be inactivating the integrated provirus, albeit through unknown mechanisms. Such mechanisms might include a shift in integration sites, inactivated provirus, and/or silencing through alterations in DNA methylation or histone modification at the HIV promoter. To support this, correlating the number of integrated copies of the provirus with the percentage of p24 producing cells following reactivation independently for pifithrin-α treated and untreated cells demonstrates parallel lines but shifted to the left for pifithrin-α treated cells (Fig 7G). Furthermore, there was a significant drop in the reactivation index between pifithrin-α treated and untreated cells (Fig 7H). These data suggest that pifithrin-α may induce changes to the integrated provirus, either directly or at the level of the epigenome, resulting in less efficient reactivation with αCD3/αCD28 beads. This is not without precedent since it has been demonstrated that HIV DNA synthesis by the virus reverse transcriptase is more accurate in the presence of p53, which has exonucleolytic proofreading capabilities [69]. Therefore, inhibition of pifithrin-α in the T_CM_ model of HIV-1 latency may lead to more error prone HIV DNA synthesis during the expansion phase resulting in greater numbers of dysfunctional provirus. Further studies of this pathway will be needed to completely understand the role of p53 in the establishment of HIV-1 latency in cultured T_CM_.

The importance of p53 signaling may not be confined to only the T_CM_ model analyzed here as several genes related to the p53 pathway were also identified in the overlap with the primary CD4 T cell model examined by Iglesias-Ussel and colleagues [9]. Specifically, the p53 related genes *ACTA2*, *BBC3*, *DDB2*, *DRAM1*, *FDXR*, *GADD45A*, and *TNFRSF10B* were significantly upregulated in both models (S2 Table). It will be interesting to determine if inhibiting the p53 pathway in other models of HIV latency also affects the establishment of latency. This may suggest common mechanisms involved across primary CD4 T cell models. Finally, the contribution of p53 to the establishment of latency *in vivo* needs to be evaluated. Interestingly, Castedo and colleagues [70] have previously shown that activation of p53 can be detected in HIV-1 infected patients in both peripheral blood mononuclear cells (PBMCs) as well lymph nodes. Moreover, the authors demonstrated that p53 activation correlates with viral load. These results suggest that p53 may also play a role in the establishment of latency *in vivo*.

The present study allowed the comparison of gene expression between the LI and UI conditions of this T_CM_ model of HIV-1 latency, which may represent potential biomarkers of latency. However, the search for these biomarkers in this study was somewhat limited by the relatively low proportion of latently infected cells in the LI condition (mean 2.92%, s.d. ±0.71%) that were in a large background of uninfected cells. Therefore, a gene must be upregulated greater than 30-fold in individual latently infected cells in order for it to be identified as being upregulated by 2-fold when comparing the LI and UI conditions. Furthermore, bystander effects could be contributing to the signal of differential gene expression between the LI and UI conditions, whereby signals emanating from previously infected cells (e.g., cytokines and chemokines) may be perturbing gene expression in uninfected cells in the LI condition. Similarly, it is plausible that these results may be confounded by the triggering of innate immune pathways in T_CM_ cells exposed but not latently infected by HIV particles. However, robust triggering of innate immune responses is unlikely, since only 11 interferon stimulated genes with known antiviral properties [71] were found to be differentially expressed between the LI and UI conditions, out of a total of 826 genes, and only 2 of these 11 genes were in the top 100 DEGs (S2 Fig). Therefore, it appears that T_CM_ cells in this model were cultured for a sufficient period of time following virus exposure to allow innate immune responses to recede. Regardless of these limitations, gene expression markers of HIV-1 latency undoubtedly exist within the 826 DEGs identified between the UI and LI conditions (S1 Table) and will need to be verified in future work.

In summary, the primary finding from this RNA-Seq analysis of the cultured T_CM_ model [20] was that p53 signaling may be involved in the establishment of HIV latency. Furthermore, the RNA-Seq data was used to demonstrate that this model was truly reflective of a latent state and thus suitable for screening latency reversing agents for shock and kill approaches to an HIV-1 cure [72,73], further investigating mechanisms associated with establishing a latent state, and identifying gene expression biomarkers of HIV-1 latency. It would be beneficial to subject all primary CD4 T cell models of HIV-1 latency [7] to RNA-Seq analysis in statistically powered studies (i.e. >3 donors) so that similarities and differences across models may be dissected. Finding similar genes across models will lead to the identification of gene expression biomarkers of HIV-1 latency that may be used to isolate latently infected cells from HIV-1 infected subjects and utilized in innovative cure strategies (e.g., radioimmunotherapy [74]) or killing latently infected cells by means of immunotoxins [75]. The profiling of other HIV-1 latency models on the omics scale will lead to the validation or modification of these models, which will undoubtedly result in a better understanding of both *in vivo* latency and the therapies that can be used to facilitate a cure for HIV.

## Methods

### Reagents

Nelfinavir was obtained through the AIDS Research and Reference Reagent Program, Division of AIDS, NIAID, NIH; raltegravir from Merck & Company, Inc.; human IL-2 from Dr. Maurice Gately, Hoffmann-La Roche Inc. [76]; HIV-1_NL4-3_ from Dr. Malcolm Martin [77]. Pifithrin-α, p-Nitro, Cyclic [78,79], a more potent analogue of pifithrin-α with a longer half-life, was obtained from Santa Cruz Biotechnology.

### Sample generation

Sample preparation and generation of infected T_CM_ cells was fully described previously [20]. Briefly, PBMCs were isolated from HIV-1 negative individuals or obtained from the Gulf Coast Regional Blood Center (Houston, TX). Naïve CD4 T cells were isolated and then activated using human αCD3/αCD28-coated magnetic beads (one bead per cell, Thermo Fisher Scientific, Cat. #11131D) in the presence of αIL-4, (1 μg/mL; Peprotech; Cat. No. 500-P24) αIL-12 (2 μg/mL; Peprotech, Cat No. 500-P154G), and tumor growth factor (TGF)-β1 (10 μg/mL; Peprotech; Cat. No. 100–21) for 3 days at a density of 500,000 cells/ml in 96 well round bottom plates. Cells were expanded in medium containing human IL-2 (30 IU/ml) for additional 4 days. IL-2 and media were replaced at day 4 and day 5. At day 7, cells were infected (or mock-infected) with HIV-1_NL4-3_ by spinoculation at 2900 rpm at 37C for 2 hours at a multiplicity of infection of 0.1. After infection, cells were further cultured in medium containing IL-2 for 3 days, subjected to crowding in round bottom plates in the presence of IL-2 for another 3 days, and then cultured for a further 4 days in the presence of IL-2 and ART in a cultured flask (nelfinavir, 0.5 μM; raltegravir, 1.0 μM). Every time that media and IL-2 were replaced, cells were kept at a density of 10^6^ cell/ml. At day 17, any remaining productively infected cells were removed by magnetic isolation of CD4+ cells using the Dynabeads CD4 Positive Isolation Kit (Thermo Fisher Scientific, Cat. No. #11551D) following the manufacturer’s instructions with a minor change, *i*.*e*. 75 μl per 10^7^ cells was used instead of 25 μl to increase the recovery of CD4 positive cells. CD4 beads were removed from the cells following the manufacture instructions. At this stage, samples were taken for the latently infected (LI) condition. Uninfected (UI) cells were cultured under the same conditions and collected at the same time as LI cells. Additional cell aliquots were subjected to reactivation with αCD3/αCD28-coated magnetic beads in the presence of ART for 2 days for the uninfected activated (UIA) and latently infected activated (LIA) conditions.

To address whether p53 transcriptional activation plays a role in the establishment of latency, 7.5 μM pifithrin-α was added 3 days post infection during the crowding stage (day 10), and then washed out with replenishment at day 13 and thus maintained in culture until just prior to reactivation (day 17) when it was washed out. Production of p24 was then assessed at day 19 after two days of reactivation with αCD3/αCD28-coated magnetic beads. In a separate experiment, to confirm that pifithrin-α was not affecting the reactivation process, pifithrin-α was added only during the reactivation step (day 17 to 19) and p24 production similarly assessed at day 19.

### RNA isolation and total RNA-seq data generation

Total RNA was extracted from 16 T_CM_ cell samples from 4 donors for the 4 conditions (UI, LI, UIA and LIA) using the RNeasy Plus Mini Kit (QIAGEN, Cat. No. 74134) according to manufacturer’s instructions, with the addition of an on-column DNase treatment (RNase-free DNase Set, QIAGEN, Cat. No. 79254). RNA integrity (RIN) values of samples were on average 9.9 (s.d. ±0.1) as determined using a Bioanalyzer 2100 (Agilent Technologies, CA, USA) and RNA concentration was measured by Nanodrop 2000 (Thermo Fisher Scientific). To account for transcriptional amplification, synthetic RNA standards from the External RNA Controls Consortium (ERCC RNA Spike-In Mix 1, Ambion, CA, USA) were spiked into total RNA isolated from each sample. T_CM_ cells in each sample were counted in quadruplicate and ERCC spike-ins were added at 1 μl of 1:100 dilution per million cells. RNA-Seq libraries were prepared from 100ng of total RNA using the TruSeq Stranded Total RNA Library Prep kit (Illumina, CA, USA) after depletion of cytoplasmic and mitochondrial ribosomal RNA with Ribo-Zero Gold (Epicentre, WI, USA). All libraries were sequenced to a read depth of >75 million reads using the Illumina HiSeq2000 to generate 50bp paired-end reads (100 bp total read length).

### Total RNA-seq data analysis

FASTQ files for each sample were mapped to the human genome (hg38) using Tophat (version 2.0.13) [80] and counted against the human GENCODE [81] annotation (v21) with HTSeq [82]. FASTQ files are available through GEO accession number (GSE81810). All reads were then aligned to the HIV_NL4-3_ genome (Genbank accession number AF324493) using TopHat and counted using HTSeq [82]. Levels of HIV-1 US, SS, and MS transcripts were estimated by the method of Mohammadi and colleagues [16] which counts reads that pass through the two major HIV-1 splice sites D1 and D4. Finally, all reads were mapped to the 92 ERCCs, using Bowtie (version 1.1.1) [83] and then counted against individual ERCCs using HTSeq. When identifying differences in host and HIV-1 gene expression between resting (UI and LI) and activated (UIA and LIA) conditions, BSN was utilized to account for transcriptional activation [26]. Briefly, the expression of ERCC spike-in controls were used to estimate a scaling factor between resting and activated conditions which was then used to adjust the expression levels of host genes and HIV-1 reads. When identifying differences in host gene expression between the UI and LI conditions, RUVSeq [84] was used to normalize the data since transcriptional amplification was not an issue in this comparison. Following normalization, differentially expressed genes were identified with EdgeR [85] (FDR corrected p-value < 0.05). Please refer to (S1 Supplemental Methods) for more details.

### Functional analysis of differentially expressed genes

Pathway analysis was performed with ToppGene [86] using the functional analysis enrichment tool, ToppFun, with the KEGG pathways selected. Pathway images were generated from the KEGG Pathway Database [30]. Fold change data were log_2_ transformed, colored, and overlaid upon the p53 signaling pathway. A protein interaction network (PIN) was generated with MetaCore and visualized through Cytoscape v2.8.3 [87]. MetaCore draws connections between the protein products of differentially expressed genes if they have protein-protein or protein-DNA interaction confirmed from the literature record. The advantage of this approach is that the PIN often reveals biological associations that have not been curated in KEGG pathways. For PIN construction, genes were filtered using a log_2_ fold change of 0.5 between latently infected and uninfected cells. Read pileup figures were generated with the Integrated Genome Browser [88]. Venn diagrams were constructed using Venny 2.1.0 [89].

### RT-qPCR validation

RT-qPCR validation of the expression of host and virus genes identified by RNA-Seq was performed using TaqMan Gene Expression Assays (Thermo Fisher Scientific) as previously described [90,91,92]. Changes in host and virus gene expression were calculated using the 2^-ΔΔCT^ method with the spike-in ERCC control ERCC_00130, as the normalizer. Please refer to (S1 Supplemental Methods) for more details on RT-qPCR analysis.

### Flow cytometry analysis

For the detection of surface FAS (CD95) expression, cells were stained with FAS/CD95 Antibody (DX2), FITC conjugate (Molecular Probes). For the detection of surface TNFRS10B (DR5) expression, cells were stained with CD262 (DR5, TRAIL-R2) Antibody (DJR2-4 (7–8)), APC conjugate (Biolegend). Cells were also stained with a viability dye (Fixable Viability Dye eFluor 450, Affymetrix, eBioscience, San Diego, CA). For the dual detection of CD4 and HIV-1 p24 Gag, cells were first stained with the viability dye (Fixable Viability Dye eFluor 450), followed by staining with CD4 antibody (S3.5), APC conjugate (Molecular Probes). After staining, cells were fixed, permeabilized, and stained for HIV-1 p24 Gag as previously described [20]. In all experiments, CD4 positive HIV-1 p24 Gag negative staining regions were set with uninfected cells treated in parallel. Flow cytometry was performed with a BD FacsCanto II flow cytometer using FACSDiva acquisition software (Becton Dickinson, Mountain View, CA). Data were analyzed with Flow Jo (TreeStar Inc, Ashland, OR).

### Measurement of HIV-1 alu PCR

DNA from 2x10^6^ cells was isolated using DNeasy Blood and Tissue Kit (Qiagen). DNA was quantified using NanoDrop 1000 (Thermo Fisher Scientific). Genomic DNA was subjected to nested quantitative Alu-LTR PCR for integrated provirus as previously described [93], with modifications. For the first reaction, 250 ng of total DNA was amplified using Platinum Taq DNA polymerase (Invitrogen). Reactions were carried with 1.5 mM of MgCl_2_, 200 μM dNTPs, 400 nM of Alu164 primer (5’-TCCCAGCTACTCGGGGAGGCTGAGG-3’) and 400 nM of PBS primer (5’-TTTCAAGTCCCTGTTCGGGCGCCA-3’). Amplifications were performed in a MultiGene Optimax (Labnet International, Inc) with the following parameters: 1) 94C 5 min; 2) 18x 94C 30 sec, 66C 30 sec, 72C 5 min; 3) 72C 10 min. PCR samples were subject to a 1/10 dilution in water, then 2 μl of the diluted sample was subject to qPCR reactions in a LightCycler 480 (Roche) using PCR Master Mix (2X) (Thermo Fisher Scientific). Final concentration of primers (AE989-2 5’-CTCTGGCTAACTAGGGAACCCAC-3’; AE990-2 5’-CTGACTAAAAGGGTCTGAGGGATCTC-3’) and probe (5’-FAM-TTAAGCCTCAATAAAGCTTGCCTTGAGTGC-BHQ1-3’) were 400 nM and 200 nM respectively. A serial dilution of pcDNA3.1-LTR was used for a molecular standard curve. pcDNA3.1-LTR was generated by cloning the 3’ LTR from NL43 into pcDNA3.1.

### Ethics statement

PBMCs were isolated from HIV-1 negative individuals following IRB-approved protocol no. #67637 (University of Utah) or unidentified source leukocytes designed “for research only” were purchased from the Gulf Coast Regional Blood Center (Houston, Texas). All HIV-1 negative individuals provided written informed consent.

## Supporting Information

S1 Fig Average RNA content increases upon activation of CD4 T cells. Total RNA was extracted from resting (UI, LI) and activated (UIA, LIA) CD4 T cells and quantified by Nanodrop. Error bars indicate standard deviation measurements across donors. Significant increase in total RNA was determined with a paired *t*-test.
(TIF)

S2 Fig Heatmap of differentially expressed genes in HIV-1 latency. Heatmap showing the top 100 differentially expressed genes (Total 826) between the LI and UI conditions based on FDR corrected *p*-values from a paired analysis. Log_2_ fold change values were used to generate this heatmap where red represents upregulation and blue downregulation according to the scale. The dendrogram represents how genes cluster based upon fold changes across donors (D1-D4) and was constructed by calculating distances using the Euclidean method and then clustering these distances with the "complete" method. Black arrows indicate antiviral interferon stimulated genes.
(TIF)

S3 Fig Cell death does not explain the lack of viral reactivation of latently infected cells generated in the presence of pifithrin-α. Cells were stained with Fixable Viability Dye eFluor 450 to determine the percentages of dead cells (**A**) with and without pifithrin-α treatment at day 13 and day 17 (pre-sorting) of culture. (**B**) Cell death was also analyzed in the CD4 positive cells after sorting to remove dead cells (day 17, post-sorting) and following stimulation with αCD3/αCD28 at day 19. Black symbols represent untreated samples and purple symbols represent cells previously treated with pifithrin-α from day 10 to 17. Each donor is presented with a different symbol. Significance was determined with a paired *t*-test (*p*-values provided).
(TIF)

S1 Table Genes differentially expressed during HIV-1 latency. A total of 826 genes were differentially expressed between the latently infected (LI) and uninfected (UI) conditions. Presented is a table of all DEGs. Log_2_FC values were calculated in EdgeR and are weighted based upon library size. Significance was determined with a FDR corrected *p*-value < 0.05. Ensembl gene IDs which did not have a corresponding gene symbol are marked as N/A.
(DOCX)

S2 Table Genes overlapping between *in vitro* models of HIV-1 latency. This table demonstrates genes which overlap in significance and direction between the T_CM_ model [20] presented in the current study and the model of Iglesias-Ussel et al. [9]. Asterisks indicate involvement with p53 signaling.
(DOCX)

S1 Supplemental Methods Supplementary methods and detailed experimental procedures. This file contains all supplemental methods for total RNA-Seq data analysis including; quantification of spliced transcripts, gene differential expression analysis, biological scaling normalization, RUVSeq normalization, as well as supplemental methods for RT-qPCR validation procedures.
(PDF)
