# Supplementary figures and images for: Transcriptomic Analysis Implicates the p53 Signaling Pathway in the Establishment of HIV-1 Latency in Central Memory CD4 T Cells in an In Vitro Model
Source: PLoS Pathog. 2016 Nov 29;12(11):e1006026. doi: 10.1371/journal.ppat.1006026 (PMC5127598; doi:10.1371/journal.ppat.1006026)

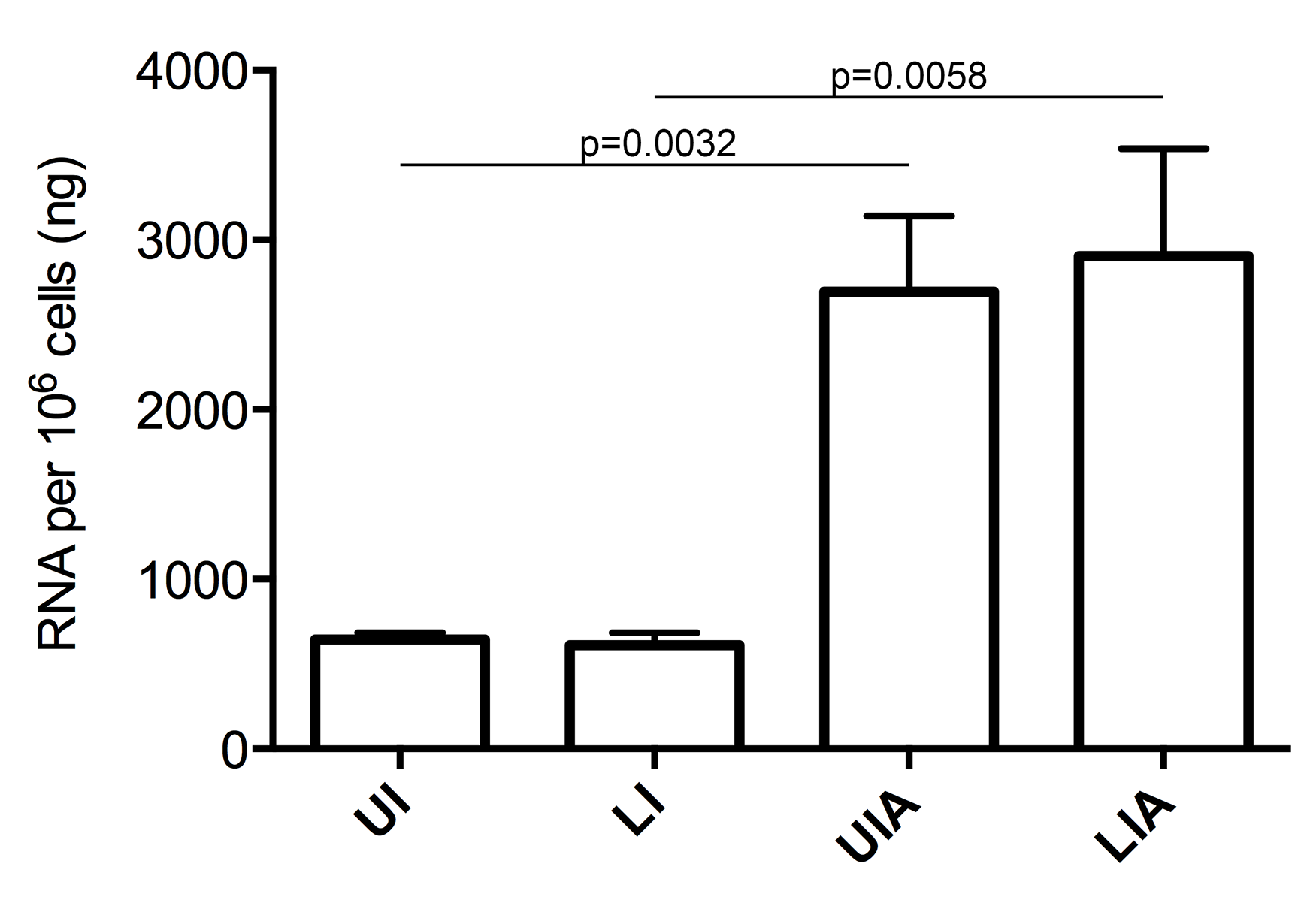

Supplement: S1 Fig — Total RNA was extracted from resting (UI, LI) and activated (UIA, LIA) CD4 T cells and quantified by Nanodrop. Error bars indicate standard deviation measurements across donors. Significant increase in total RNA was determined with a paired t-test. (TIF) [file ppat.1006026.s001.tif]

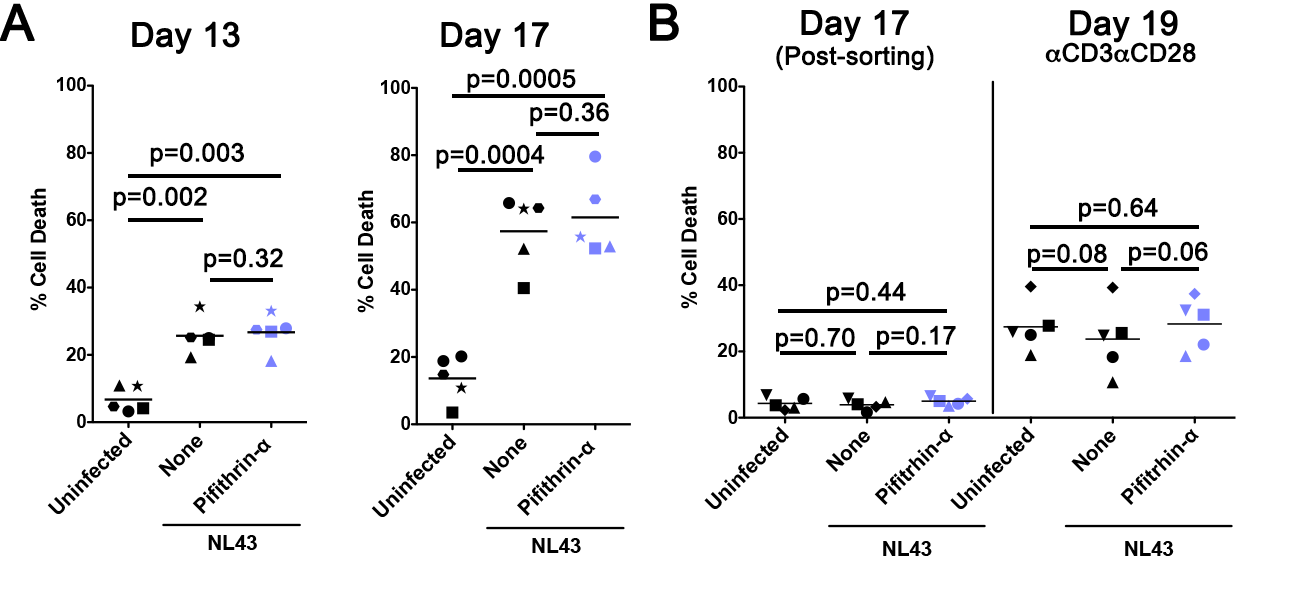

Supplement: S3 Fig — Cells were stained with Fixable Viability Dye eFluor 450 to determine the percentages of dead cells (A) with and without pifithrin-α treatment at day 13 and day 17 (pre-sorting) of culture. (B) Cell death was also analyzed in the CD4 positive cells after sorting to remove dead cells (day 17, post-sorting) and following stimulation with αCD3/αCD28 at day 19. Black symbols represent untreated samples and purple symbols represent cells previously treated with pifithrin-α from day 10 to 17. Each donor is presented with a different symbol. Significance was determined with a paired t-test (p-values provided). (TIF) [file ppat.1006026.s003.tif]
