# Supplementary material for: Transcriptomic Analysis Implicates the p53 Signaling Pathway in the Establishment of HIV-1 Latency in Central Memory CD4 T Cells in an In Vitro Model
Source: PLoS Pathog. 2016 Nov 29;12(11):e1006026. doi: 10.1371/journal.ppat.1006026 (PMC5127598; doi:10.1371/journal.ppat.1006026)
Supplement: S1 Table — A total of 826 genes were differentially expressed between the latently infected (LI) and uninfected (UI) conditions. Presented is a table of all DEGs. Log2FC values were calculated in EdgeR and are weighted based upon library size. Significance was determined with a FDR corrected p-value < 0.05. Ensembl gene IDs which did not have a corresponding gene symbol are marked as N/A. (DOCX) [file ppat.1006026.s004.docx]

| **Ensembl ID** | **Gene Name** | **Log_2_FC** | **FDR** |
| --- | --- | --- | --- |
| ENSG00000174307 | PHLDA3 | 1.1578 | 6.85E-24 |
| ENSG00000064886 | CHI3L2 | 0.9489 | 8.39E-19 |
| ENSG00000031081 | ARHGAP31 | 1.4814 | 1.87E-17 |
| ENSG00000020577 | SAMD4A | 1.0188 | 7.25E-15 |
| ENSG00000161513 | FDXR | 1.0786 | 4.20E-14 |
| ENSG00000181026 | AEN | 0.6627 | 6.15E-14 |
| ENSG00000139629 | GALNT6 | 1.0517 | 7.67E-14 |
| ENSG00000142798 | HSPG2 | 1.5276 | 1.39E-13 |
| ENSG00000136048 | DRAM1 | 1.0252 | 5.51E-13 |
| ENSG00000100298 | APOBEC3H | 1.5041 | 1.25E-11 |
| ENSG00000196814 | MVB12B | 1.6640 | 2.23E-11 |
| ENSG00000164237 | CMBL | 1.9720 | 2.23E-11 |
| ENSG00000025434 | NR1H3 | 1.2118 | 3.65E-11 |
| ENSG00000116717 | GADD45A | 0.9441 | 1.05E-10 |
| ENSG00000148219 | ASTN2 | 1.0110 | 1.05E-10 |
| ENSG00000104870 | FCGRT | 0.8420 | 1.73E-09 |
| ENSG00000143126 | CELSR2 | 0.7973 | 1.73E-09 |
| ENSG00000234546 | RP3-510D11.2 | 1.2151 | 2.02E-09 |
| ENSG00000160179 | ABCG1 | 1.0039 | 3.15E-09 |
| ENSG00000170899 | GSTA4 | 1.4965 | 5.91E-09 |
| ENSG00000172667 | ZMAT3 | 0.5554 | 1.73E-08 |
| ENSG00000239636 | RP4-728D4.2 | 1.1281 | 1.73E-08 |
| ENSG00000107796 | ACTA2 | 1.0234 | 1.95E-08 |
| ENSG00000135919 | SERPINE2 | 1.3184 | 2.73E-08 |
| ENSG00000233056 | ERVH48-1 | -1.4125 | 3.84E-08 |
| ENSG00000009694 | TENM1 | -1.2523 | 3.97E-08 |
| ENSG00000272654 | RP11-422P24.11 | 1.1199 | 4.67E-08 |
| ENSG00000166548 | TK2 | 0.6549 | 5.44E-08 |
| ENSG00000080561 | MID2 | 0.6349 | 6.81E-08 |
| ENSG00000269900 | RMRP | -0.5783 | 7.80E-08 |
| ENSG00000169398 | PTK2 | 1.6135 | 1.75E-07 |
| ENSG00000121742 | GJB6 | 0.7907 | 2.55E-07 |
| ENSG00000108932 | SLC16A6 | 1.0151 | 2.76E-07 |
| ENSG00000145491 | ROPN1L | 1.2599 | 3.46E-07 |
| ENSG00000065361 | ERBB3 | 0.8686 | 4.28E-07 |
| ENSG00000134247 | PTGFRN | 1.0809 | 6.29E-07 |
| ENSG00000153936 | HS2ST1 | 0.5074 | 6.44E-07 |
| ENSG00000110002 | VWA5A | 0.5465 | 6.44E-07 |
| ENSG00000171097 | CCBL1 | 0.6324 | 6.44E-07 |
| ENSG00000204396 | VWA7 | 1.6046 | 6.87E-07 |
| ENSG00000171208 | NETO2 | -1.2523 | 8.81E-07 |
| ENSG00000057657 | PRDM1 | 0.8358 | 1.29E-06 |
| ENSG00000077782 | FGFR1 | 0.6705 | 1.30E-06 |
| ENSG00000164050 | PLXNB1 | 1.4033 | 1.87E-06 |
| ENSG00000261504 | RP11-317P15.4 | 1.6055 | 2.14E-06 |
| ENSG00000111254 | AKAP3 | 0.7896 | 2.30E-06 |
| ENSG00000135423 | GLS2 | 1.4279 | 3.15E-06 |
| ENSG00000157077 | ZFYVE9 | 0.6307 | 4.34E-06 |
| ENSG00000173334 | TRIB1 | 0.7251 | 5.42E-06 |
| ENSG00000233093 | LINC00892 | -1.1709 | 6.93E-06 |
| ENSG00000117586 | TNFSF4 | 1.2178 | 6.94E-06 |
| ENSG00000175938 | ORAI3 | 0.6466 | 7.08E-06 |
| ENSG00000260193 | RP11-83N9.5 | 1.0685 | 7.64E-06 |
| ENSG00000181104 | F2R | 1.0196 | 8.69E-06 |
| ENSG00000135679 | MDM2 | 0.5876 | 8.69E-06 |
| ENSG00000101400 | SNTA1 | 0.6720 | 8.78E-06 |
| ENSG00000185088 | RPS27L | 0.4643 | 9.02E-06 |
| ENSG00000178607 | ERN1 | -0.4946 | 9.02E-06 |
| ENSG00000162843 | WDR64 | 1.1023 | 9.02E-06 |
| ENSG00000120889 | TNFRSF10B | 0.5114 | 1.01E-05 |
| ENSG00000197852 | FAM212B | 0.7304 | 1.06E-05 |
| ENSG00000115607 | IL18RAP | 0.8428 | 1.30E-05 |
| ENSG00000269343 | ZNF587B | -0.5572 | 1.43E-05 |
| ENSG00000060558 | GNA15 | 0.8606 | 1.50E-05 |
| ENSG00000026103 | FAS | 0.5407 | 1.53E-05 |
| ENSG00000214189 | ZNF788 | 0.9492 | 2.16E-05 |
| ENSG00000224470 | ATXN1L | -0.4531 | 2.32E-05 |
| ENSG00000136295 | TTYH3 | 0.5548 | 2.38E-05 |
| ENSG00000273749 | CYFIP1 | 0.5631 | 2.39E-05 |
| ENSG00000170836 | PPM1D | 0.4753 | 2.44E-05 |
| ENSG00000100439 | ABHD4 | 0.5141 | 2.52E-05 |
| ENSG00000131080 | EDA2R | 0.5771 | 2.77E-05 |
| ENSG00000272325 | NUDT3 | -0.4881 | 2.85E-05 |
| ENSG00000230487 | PSMG3-AS1 | 0.6301 | 3.09E-05 |
| ENSG00000186834 | HEXIM1 | -0.5355 | 3.25E-05 |
| ENSG00000092096 | SLC22A17 | 0.8836 | 3.46E-05 |
| ENSG00000185432 | METTL7A | 0.8310 | 3.62E-05 |
| ENSG00000143801 | PSEN2 | 0.6332 | 3.95E-05 |
| ENSG00000196866 | HIST1H2AD | -0.4444 | 3.95E-05 |
| ENSG00000223820 | CFL1P1 | 0.9189 | 4.10E-05 |
| ENSG00000134574 | DDB2 | 0.4983 | 4.18E-05 |
| ENSG00000108852 | MPP2 | 1.5830 | 4.89E-05 |
| ENSG00000167904 | TMEM68 | 0.4267 | 4.94E-05 |
| ENSG00000260912 | RP11-363E7.4 | 0.8193 | 5.01E-05 |
| ENSG00000132646 | PCNA | 0.4411 | 5.01E-05 |
| ENSG00000119471 | HSDL2 | 0.3768 | 5.43E-05 |
| ENSG00000251301 | RP11-81H14.2 | 1.0696 | 5.90E-05 |
| ENSG00000277161 | PIGW | -0.4379 | 5.90E-05 |
| ENSG00000100285 | NEFH | -1.2687 | 5.99E-05 |
| ENSG00000122694 | GLIPR2 | 0.6478 | 6.33E-05 |
| ENSG00000052749 | RRP12 | -0.4682 | 6.82E-05 |
| ENSG00000160447 | PKN3 | 1.0046 | 6.94E-05 |
| ENSG00000163071 | SPATA18 | 1.5635 | 8.67E-05 |
| ENSG00000010278 | CD9 | 0.8893 | 8.67E-05 |
| ENSG00000274922 | RP11-88E10.5 | 0.6113 | 1.03E-04 |
| ENSG00000143344 | RGL1 | 0.6231 | 1.16E-04 |
| ENSG00000168734 | PKIG | -0.8192 | 1.23E-04 |
| ENSG00000267390 | RP11-635N19.1 | 0.7511 | 1.51E-04 |
| ENSG00000167695 | FAM57A | 0.8201 | 1.51E-04 |
| ENSG00000127952 | STYXL1 | 0.4003 | 1.52E-04 |
| ENSG00000196562 | SULF2 | 2.2991 | 2.09E-04 |
| ENSG00000054148 | PHPT1 | 0.5141 | 2.10E-04 |
| ENSG00000054523 | KIF1B | 0.4689 | 2.22E-04 |
| ENSG00000138722 | MMRN1 | 1.0260 | 2.25E-04 |
| ENSG00000145649 | GZMA | 1.0969 | 2.28E-04 |
| ENSG00000071246 | VASH1 | 0.6612 | 2.31E-04 |
| ENSG00000198400 | NTRK1 | 1.0569 | 2.51E-04 |
| ENSG00000243176 | RP11-550I24.2 | 0.7054 | 2.63E-04 |
| ENSG00000078237 | C12orf5 | 0.4874 | 2.78E-04 |
| ENSG00000225889 | AC074289.1 | 0.9607 | 2.81E-04 |
| ENSG00000105327 | BBC3 | 0.5515 | 2.86E-04 |
| ENSG00000155366 | RHOC | 0.5795 | 2.90E-04 |
| ENSG00000134072 | CAMK1 | 0.7815 | 3.01E-04 |
| ENSG00000105559 | PLEKHA4 | 0.7070 | 3.02E-04 |
| ENSG00000153363 | LINC00467 | 0.6412 | 3.02E-04 |
| ENSG00000124145 | SDC4 | 0.9998 | 3.02E-04 |
| ENSG00000196141 | SPATS2L | 0.6245 | 3.02E-04 |
| ENSG00000101546 | RBFA | -0.4768 | 3.20E-04 |
| ENSG00000047230 | CTPS2 | 0.5403 | 3.24E-04 |
| ENSG00000011638 | TMEM159 | 0.5141 | 3.39E-04 |
| ENSG00000270959 | LPP-AS2 | 0.6572 | 4.14E-04 |
| ENSG00000154263 | ABCA10 | 0.7997 | 4.18E-04 |
| ENSG00000160683 | CXCR5 | 1.0520 | 4.23E-04 |
| ENSG00000120256 | LRP11 | 0.6840 | 4.68E-04 |
| ENSG00000197879 | MYO1C | 0.4518 | 5.05E-04 |
| ENSG00000005243 | COPZ2 | 0.9719 | 5.19E-04 |
| ENSG00000158321 | AUTS2 | -1.1106 | 5.52E-04 |
| ENSG00000148057 | IDNK | 0.4702 | 5.53E-04 |
| ENSG00000165185 | KIAA1958 | 0.5276 | 5.58E-04 |
| ENSG00000161921 | CXCL16 | 1.0184 | 5.65E-04 |
| ENSG00000204161 | C10orf128 | -0.7344 | 5.98E-04 |
| ENSG00000161533 | ACOX1 | 0.3379 | 6.27E-04 |
| ENSG00000213719 | CLIC1 | 0.4160 | 6.27E-04 |
| ENSG00000164400 | CSF2 | -1.7184 | 6.28E-04 |
| ENSG00000108352 | RAPGEFL1 | 0.8841 | 6.28E-04 |
| ENSG00000131634 | TMEM204 | -0.4914 | 6.65E-04 |
| ENSG00000268006 | PTOV1-AS1 | -0.8465 | 6.66E-04 |
| ENSG00000120306 | CYSTM1 | 0.4616 | 6.66E-04 |
| ENSG00000171067 | C11orf24 | 0.5143 | 6.99E-04 |
| ENSG00000081181 | ARG2 | -0.8120 | 7.40E-04 |
| ENSG00000232533 | AC093673.5 | 0.6653 | 7.65E-04 |
| ENSG00000132879 | FBXO44 | 0.4526 | 7.65E-04 |
| ENSG00000272476 | RP1-191J18.66 | -0.7695 | 7.68E-04 |
| ENSG00000272758 | RP11-299J3.8 | 0.4986 | 7.68E-04 |
| ENSG00000115129 | TP53I3 | 1.0278 | 7.70E-04 |
| ENSG00000197965 | MPZL1 | 0.4973 | 7.78E-04 |
| ENSG00000180694 | TMEM64 | 0.5152 | 8.23E-04 |
| ENSG00000152465 | NMT2 | 0.3373 | 8.64E-04 |
| ENSG00000169692 | AGPAT2 | 0.5265 | 8.64E-04 |
| ENSG00000113328 | CCNG1 | 0.4725 | 8.70E-04 |
| ENSG00000225079 | FTH1P22 | 0.9095 | 8.76E-04 |
| ENSG00000105519 | CAPS | -0.5642 | 9.30E-04 |
| ENSG00000148450 | MSRB2 | 0.7058 | 9.89E-04 |
| ENSG00000154767 | XPC | 0.4777 | 9.89E-04 |
| ENSG00000204472 | AIF1 | -0.7562 | 1.00E-03 |
| ENSG00000143479 | DYRK3 | -0.7969 | 1.06E-03 |
| ENSG00000173451 | THAP2 | 0.4650 | 1.06E-03 |
| ENSG00000102265 | TIMP1 | 0.5729 | 1.12E-03 |
| ENSG00000135925 | WNT10A | 1.3565 | 1.12E-03 |
| ENSG00000107954 | NEURL1 | -1.0588 | 1.14E-03 |
| ENSG00000167554 | ZNF610 | 1.0048 | 1.15E-03 |
| ENSG00000150756 | FAM173B | 0.4455 | 1.16E-03 |
| ENSG00000136997 | MYC | -0.4974 | 1.19E-03 |
| ENSG00000138119 | MYOF | -1.6840 | 1.19E-03 |
| ENSG00000173715 | C11orf80 | 0.5124 | 1.21E-03 |
| ENSG00000148053 | NTRK2 | -1.8290 | 1.23E-03 |
| ENSG00000163053 | SLC16A14 | 2.3647 | 1.23E-03 |
| ENSG00000118257 | NRP2 | -0.5500 | 1.23E-03 |
| ENSG00000103196 | CRISPLD2 | -0.7638 | 1.25E-03 |
| ENSG00000132170 | PPARG | -3.2256 | 1.33E-03 |
| ENSG00000172456 | FGGY | 0.5173 | 1.33E-03 |
| ENSG00000151116 | UEVLD | 0.3959 | 1.33E-03 |
| ENSG00000171467 | ZNF318 | 0.3893 | 1.36E-03 |
| ENSG00000040199 | PHLPP2 | 0.4024 | 1.39E-03 |
| ENSG00000253878 | RP11-347C18.3 | 0.7320 | 1.39E-03 |
| ENSG00000188177 | ZC3H6 | 0.3666 | 1.39E-03 |
| ENSG00000108106 | UBE2S | -0.4872 | 1.41E-03 |
| ENSG00000186951 | PPARA | 0.3801 | 1.43E-03 |
| ENSG00000198910 | L1CAM | 0.5987 | 1.57E-03 |
| ENSG00000213722 | DDAH2 | 0.6947 | 1.57E-03 |
| ENSG00000134070 | IRAK2 | 0.4846 | 1.62E-03 |
| ENSG00000148840 | PPRC1 | -0.3546 | 1.62E-03 |
| ENSG00000100983 | GSS | 0.3398 | 1.62E-03 |
| ENSG00000174514 | MFSD4 | 0.6199 | 1.62E-03 |
| ENSG00000187475 | HIST1H1T | -2.3801 | 1.64E-03 |
| ENSG00000168785 | TSPAN5 | 0.4097 | 1.66E-03 |
| ENSG00000244509 | APOBEC3C | 0.3656 | 1.67E-03 |
| ENSG00000105447 | GRWD1 | -0.4280 | 1.67E-03 |
| ENSG00000165029 | ABCA1 | 3.3768 | 1.67E-03 |
| ENSG00000240038 | AMY2B | 0.6194 | 1.67E-03 |
| ENSG00000277075 | HIST1H2AE | -0.3729 | 1.75E-03 |
| ENSG00000102897 | LYRM1 | 0.4341 | 1.79E-03 |
| ENSG00000198933 | TBKBP1 | 0.5630 | 1.79E-03 |
| ENSG00000169194 | IL13 | -2.5274 | 1.79E-03 |
| ENSG00000164742 | ADCY1 | -3.2545 | 1.85E-03 |
| ENSG00000163389 | POGLUT1 | -0.4876 | 1.85E-03 |
| ENSG00000157483 | MYO1E | 0.6217 | 1.89E-03 |
| ENSG00000165886 | UBTD1 | 0.7642 | 1.89E-03 |
| ENSG00000130518 | KIAA1683 | -0.8258 | 1.90E-03 |
| ENSG00000232208 | RP3-477M7.5 | 0.8385 | 2.03E-03 |
| ENSG00000070526 | ST6GALNAC1 | -0.7572 | 2.03E-03 |
| ENSG00000120656 | TAF12 | 0.3652 | 2.06E-03 |
| ENSG00000102471 | NDFIP2 | 0.6177 | 2.12E-03 |
| ENSG00000153982 | GDPD1 | 0.6965 | 2.12E-03 |
| ENSG00000124570 | SERPINB6 | 0.5270 | 2.12E-03 |
| ENSG00000137842 | TMEM62 | 0.5476 | 2.21E-03 |
| ENSG00000095209 | TMEM38B | 0.3618 | 2.35E-03 |
| ENSG00000107815 | C10orf2 | -0.4406 | 2.35E-03 |
| ENSG00000125388 | GRK4 | 0.5489 | 2.37E-03 |
| ENSG00000228439 | TSTD3 | 0.7772 | 2.41E-03 |
| ENSG00000137831 | UACA | 1.3815 | 2.44E-03 |
| ENSG00000177674 | AGTRAP | 0.4846 | 2.45E-03 |
| ENSG00000134765 | DSC1 | -1.2594 | 2.45E-03 |
| ENSG00000048162 | NOP16 | -0.4696 | 2.52E-03 |
| ENSG00000116663 | FBXO6 | 0.5344 | 2.59E-03 |
| ENSG00000010318 | PHF7 | 0.4400 | 2.59E-03 |
| ENSG00000146834 | MEPCE | -0.4443 | 2.68E-03 |
| ENSG00000161547 | SRSF2 | -0.3246 | 2.68E-03 |
| ENSG00000104907 | TRMT1 | -0.3677 | 2.70E-03 |
| ENSG00000136720 | HS6ST1 | 0.3879 | 2.71E-03 |
| ENSG00000101003 | GINS1 | 0.5778 | 2.82E-03 |
| ENSG00000196981 | WDR5B | -0.3715 | 2.82E-03 |
| ENSG00000111796 | KLRB1 | -0.7332 | 2.82E-03 |
| ENSG00000273061 | CDC37L1-AS1 | 0.6727 | 2.86E-03 |
| ENSG00000177076 | ACER2 | 0.5365 | 2.93E-03 |
| ENSG00000180263 | FGD6 | 0.9039 | 2.95E-03 |
| ENSG00000249859 | PVT1 | 0.3672 | 3.02E-03 |
| ENSG00000086015 | MAST2 | 0.4834 | 3.03E-03 |
| ENSG00000198919 | DZIP3 | 0.4346 | 3.04E-03 |
| ENSG00000135931 | ARMC9 | -1.7910 | 3.05E-03 |
| ENSG00000273729 | RP11-7F17.8 | 0.6762 | 3.11E-03 |
| ENSG00000115641 | FHL2 | 0.8505 | 3.22E-03 |
| ENSG00000232855 | AF131217.1 | 0.6535 | 3.31E-03 |
| ENSG00000146904 | EPHA1 | -1.0996 | 3.31E-03 |
| ENSG00000124067 | SLC12A4 | 0.3785 | 3.33E-03 |
| ENSG00000163806 | SPDYA | -0.7037 | 3.33E-03 |
| ENSG00000128394 | APOBEC3F | 0.5412 | 3.33E-03 |
| ENSG00000137166 | FOXP4 | 0.9886 | 3.33E-03 |
| ENSG00000119139 | TJP2 | 0.4351 | 3.33E-03 |
| ENSG00000111666 | CHPT1 | 0.5271 | 3.33E-03 |
| ENSG00000236935 | AP003774.1 | 0.5951 | 3.38E-03 |
| ENSG00000204866 | IGFL2 | -4.8534 | 3.45E-03 |
| ENSG00000167306 | MYO5B | 1.3784 | 3.57E-03 |
| ENSG00000120278 | PLEKHG1 | 0.9806 | 3.61E-03 |
| ENSG00000035720 | STAP1 | 0.6832 | 3.61E-03 |
| ENSG00000128524 | ATP6V1F | 0.3086 | 3.64E-03 |
| ENSG00000165424 | ZCCHC24 | 0.8282 | 3.66E-03 |
| ENSG00000177410 | ZFAS1 | -0.3611 | 3.67E-03 |
| ENSG00000172794 | RAB37 | 0.4028 | 3.67E-03 |
| ENSG00000006756 | ARSD | 0.4668 | 3.67E-03 |
| ENSG00000254965 | RP11-113K21.2 | 0.6128 | 3.67E-03 |
| ENSG00000198734 | F5 | 0.6841 | 3.67E-03 |
| ENSG00000145777 | TSLP | 1.2684 | 3.67E-03 |
| ENSG00000247853 | RP5-940J5.6 | 0.7817 | 3.67E-03 |
| ENSG00000163923 | RPL39L | 0.7995 | 3.68E-03 |
| ENSG00000275464 | CH507-9B2.5 | -0.5536 | 3.70E-03 |
| ENSG00000225511 | LINC00475 | 1.7488 | 3.76E-03 |
| ENSG00000257315 | ZBED6 | -0.3449 | 3.79E-03 |
| ENSG00000171462 | DLK2 | 1.6754 | 3.90E-03 |
| ENSG00000132274 | TRIM22 | 0.3337 | 3.90E-03 |
| ENSG00000188157 | AGRN | 0.7197 | 3.91E-03 |
| ENSG00000250903 | GMDS-AS1 | 0.4181 | 3.97E-03 |
| ENSG00000272053 | RP11-367G6.3 | 0.7816 | 4.03E-03 |
| ENSG00000273338 | RP11-386I14.4 | -0.6988 | 4.03E-03 |
| ENSG00000135596 | MICAL1 | 0.3211 | 4.04E-03 |
| ENSG00000187860 | CCDC157 | -0.7193 | 4.04E-03 |
| ENSG00000171298 | GAA | 0.3670 | 4.04E-03 |
| ENSG00000113739 | STC2 | -0.9739 | 4.10E-03 |
| ENSG00000162775 | RBM15 | -0.3027 | 4.14E-03 |
| ENSG00000085719 | CPNE3 | 0.3061 | 4.15E-03 |
| ENSG00000170917 | NUDT6 | 0.5766 | 4.30E-03 |
| ENSG00000274929 | RP11-165D7.5 | 0.7305 | 4.37E-03 |
| ENSG00000174010 | KLHL15 | -0.3853 | 4.41E-03 |
| ENSG00000065485 | PDIA5 | -0.5487 | 4.41E-03 |
| ENSG00000259994 | RP11-305E6.4 | -0.7949 | 4.48E-03 |
| ENSG00000176393 | RNPEP | 0.3165 | 4.48E-03 |
| ENSG00000107968 | MAP3K8 | 0.5517 | 4.62E-03 |
| ENSG00000198816 | ZNF358 | 0.5286 | 4.62E-03 |
| ENSG00000213563 | C8orf82 | -0.3725 | 4.68E-03 |
| ENSG00000111845 | PAK1IP1 | -0.3374 | 4.82E-03 |
| ENSG00000083290 | ULK2 | 0.5203 | 5.05E-03 |
| ENSG00000100628 | ASB2 | 0.5252 | 5.07E-03 |
| ENSG00000140678 | ITGAX | 0.9973 | 5.09E-03 |
| ENSG00000234614 | AL450992.2 | 0.6846 | 5.12E-03 |
| ENSG00000259065 | RP5-1021I20.1 | 0.6520 | 5.14E-03 |
| ENSG00000169047 | IRS1 | 0.4845 | 5.15E-03 |
| ENSG00000242474 | AC093627.9 | -0.7940 | 5.16E-03 |
| ENSG00000155265 | GOLGA7B | -0.5923 | 5.16E-03 |
| ENSG00000259943 | RP1-39G22.7 | 0.4086 | 5.24E-03 |
| ENSG00000267731 | RP11-147L13.8 | 0.6873 | 5.24E-03 |
| ENSG00000174928 | C3orf33 | 0.5166 | 5.29E-03 |
| ENSG00000214706 | IFRD2 | -0.3172 | 5.32E-03 |
| ENSG00000177406 | RP11-218M22.1 | 0.4915 | 5.57E-03 |
| ENSG00000149577 | SIDT2 | 0.3566 | 5.67E-03 |
| ENSG00000143365 | RORC | -1.0365 | 5.67E-03 |
| ENSG00000145284 | SCD5 | 1.6206 | 5.67E-03 |
| ENSG00000079257 | LXN | 0.4012 | 5.67E-03 |
| ENSG00000178852 | EFCAB13 | 0.5088 | 5.77E-03 |
| ENSG00000228485 | GRK5-IT1 | -0.7890 | 5.78E-03 |
| ENSG00000111684 | LPCAT3 | 0.3827 | 5.78E-03 |
| ENSG00000105204 | DYRK1B | -0.4743 | 5.79E-03 |
| ENSG00000259436 | CTC-378H22.2 | 0.5493 | 5.98E-03 |
| ENSG00000185924 | RTN4RL1 | 0.7065 | 6.06E-03 |
| ENSG00000273802 | HIST1H2BG | -0.3427 | 6.11E-03 |
| ENSG00000090447 | TFAP4 | -0.4281 | 6.11E-03 |
| ENSG00000126351 | THRA | 0.5604 | 6.11E-03 |
| ENSG00000141198 | TOM1L1 | -3.4594 | 6.20E-03 |
| ENSG00000171444 | MCC | 0.8659 | 6.28E-03 |
| ENSG00000164048 | ZNF589 | -0.3421 | 6.48E-03 |
| ENSG00000155093 | PTPRN2 | 0.8745 | 6.76E-03 |
| ENSG00000165410 | CFL2 | 0.6623 | 6.80E-03 |
| ENSG00000176485 | PLA2G16 | 0.9575 | 6.81E-03 |
| ENSG00000196793 | ZNF239 | -1.1356 | 6.86E-03 |
| ENSG00000267350 | N/A | -0.3231 | 6.96E-03 |
| ENSG00000241343 | RPL36A | -0.5061 | 6.96E-03 |
| ENSG00000182224 | CYB5D1 | -0.5941 | 7.10E-03 |
| ENSG00000163584 | RPL22L1 | -0.3691 | 7.13E-03 |
| ENSG00000268942 | CKS1BP3 | 1.4213 | 7.13E-03 |
| ENSG00000134030 | CTIF | 0.8471 | 7.18E-03 |
| ENSG00000167470 | MIDN | -0.4116 | 7.22E-03 |
| ENSG00000115758 | ODC1 | -0.3196 | 7.22E-03 |
| ENSG00000196218 | RYR1 | 0.9104 | 7.22E-03 |
| ENSG00000164674 | SYTL3 | 0.5991 | 7.51E-03 |
| ENSG00000189050 | RNFT1 | 0.3635 | 7.71E-03 |
| ENSG00000084207 | GSTP1 | 0.3649 | 7.78E-03 |
| ENSG00000169429 | CXCL8 | -2.7052 | 7.82E-03 |
| ENSG00000184270 | HIST2H2AB | -0.3955 | 7.82E-03 |
| ENSG00000109501 | WFS1 | 0.6228 | 7.85E-03 |
| ENSG00000119280 | C1orf198 | 0.6052 | 7.95E-03 |
| ENSG00000166228 | PCBD1 | 0.5246 | 7.96E-03 |
| ENSG00000168807 | SNTB2 | 0.3894 | 8.18E-03 |
| ENSG00000127526 | SLC35E1 | 0.3086 | 8.28E-03 |
| ENSG00000114942 | EEF1B2 | -0.3882 | 8.37E-03 |
| ENSG00000166669 | ATF7IP2 | 0.3034 | 8.50E-03 |
| ENSG00000104356 | POP1 | -0.4134 | 8.50E-03 |
| ENSG00000132768 | DPH2 | -0.3351 | 8.50E-03 |
| ENSG00000172465 | TCEAL1 | 0.4874 | 8.50E-03 |
| ENSG00000166262 | FAM227B | 0.4626 | 8.68E-03 |
| ENSG00000143067 | ZNF697 | -1.6412 | 8.77E-03 |
| ENSG00000108256 | NUFIP2 | -0.3322 | 9.03E-03 |
| ENSG00000176124 | DLEU1 | -0.3413 | 9.03E-03 |
| ENSG00000120800 | UTP20 | -0.3043 | 9.13E-03 |
| ENSG00000137965 | IFI44 | 0.7024 | 9.23E-03 |
| ENSG00000181873 | IBA57 | -0.3694 | 9.23E-03 |
| ENSG00000198393 | ZNF26 | -0.3454 | 9.31E-03 |
| ENSG00000128604 | IRF5 | 0.6306 | 9.41E-03 |
| ENSG00000197497 | ZNF665 | 0.6545 | 9.41E-03 |
| ENSG00000140043 | PTGR2 | 0.6632 | 9.41E-03 |
| ENSG00000132359 | RAP1GAP2 | 0.5108 | 9.52E-03 |
| ENSG00000176714 | CCDC121 | -0.7725 | 9.53E-03 |
| ENSG00000102804 | TSC22D1 | 0.3682 | 9.54E-03 |
| ENSG00000165175 | MID1IP1 | -0.3153 | 9.59E-03 |
| ENSG00000115183 | TANC1 | 1.1835 | 9.72E-03 |
| ENSG00000277879 | RP11-129M16.4 | -0.7526 | 9.79E-03 |
| ENSG00000110756 | HPS5 | 0.3138 | 9.81E-03 |
| ENSG00000236814 | RP11-446E9.1 | -0.7278 | 9.81E-03 |
| ENSG00000254986 | DPP3 | -0.3171 | 9.84E-03 |
| ENSG00000179988 | PSTK | -0.4710 | 9.84E-03 |
| ENSG00000162073 | PAQR4 | 0.3870 | 9.84E-03 |
| ENSG00000164976 | KIAA1161 | -0.8311 | 9.88E-03 |
| ENSG00000163811 | WDR43 | -0.2683 | 1.00E-02 |
| ENSG00000271288 | IGHV1OR15-3 | 1.0955 | 1.00E-02 |
| ENSG00000126803 | HSPA2 | -0.6918 | 1.00E-02 |
| ENSG00000116560 | SFPQ | -0.2786 | 1.00E-02 |
| ENSG00000105246 | EBI3 | -1.3550 | 1.01E-02 |
| ENSG00000161179 | YDJC | -0.3904 | 1.03E-02 |
| ENSG00000166398 | KIAA0355 | 0.3303 | 1.03E-02 |
| ENSG00000146376 | ARHGAP18 | 0.4302 | 1.03E-02 |
| ENSG00000128791 | TWSG1 | 0.4167 | 1.03E-02 |
| ENSG00000128342 | LIF | 0.6559 | 1.04E-02 |
| ENSG00000126458 | RRAS | 0.4253 | 1.05E-02 |
| ENSG00000257261 | RP11-96H19.1 | 0.6675 | 1.05E-02 |
| ENSG00000197959 | DNM3 | 0.6088 | 1.06E-02 |
| ENSG00000272016 | N/A | 0.8661 | 1.07E-02 |
| ENSG00000163599 | CTLA4 | 0.4550 | 1.09E-02 |
| ENSG00000173530 | TNFRSF10D | 0.7597 | 1.09E-02 |
| ENSG00000114439 | BBX | 0.3411 | 1.10E-02 |
| ENSG00000163110 | PDLIM5 | 0.3383 | 1.10E-02 |
| ENSG00000064115 | TM7SF3 | 0.3781 | 1.10E-02 |
| ENSG00000260260 | SNHG19 | -0.7007 | 1.11E-02 |
| ENSG00000261351 | CTD-3185P2.1 | -0.5428 | 1.13E-02 |
| ENSG00000160226 | C21orf2 | -0.5023 | 1.13E-02 |
| ENSG00000108561 | C1QBP | -0.3123 | 1.15E-02 |
| ENSG00000109684 | CLNK | 2.1398 | 1.15E-02 |
| ENSG00000163590 | PPM1L | 0.4152 | 1.15E-02 |
| ENSG00000102317 | RBM3 | -0.3758 | 1.15E-02 |
| ENSG00000173727 | CMB9-22P13.1 | 0.8577 | 1.15E-02 |
| ENSG00000105982 | RNF32 | 0.6469 | 1.15E-02 |
| ENSG00000105849 | TWISTNB | -0.3072 | 1.15E-02 |
| ENSG00000158373 | HIST1H2BD | -0.3558 | 1.16E-02 |
| ENSG00000148832 | PAOX | 0.5738 | 1.18E-02 |
| ENSG00000224429 | LINC00539 | -0.8394 | 1.19E-02 |
| ENSG00000243323 | PTPRVP | 0.8000 | 1.20E-02 |
| ENSG00000078081 | LAMP3 | 0.8910 | 1.21E-02 |
| ENSG00000167535 | CACNB3 | 0.6985 | 1.22E-02 |
| ENSG00000134202 | GSTM3 | 0.5177 | 1.24E-02 |
| ENSG00000225632 | RP5-997D24.3 | -0.6156 | 1.25E-02 |
| ENSG00000077713 | SLC25A43 | 0.3754 | 1.25E-02 |
| ENSG00000125510 | OPRL1 | 0.7419 | 1.25E-02 |
| ENSG00000188483 | IER5L | -0.7197 | 1.26E-02 |
| ENSG00000186994 | KANK3 | 0.6486 | 1.28E-02 |
| ENSG00000185745 | IFIT1 | 1.0740 | 1.28E-02 |
| ENSG00000127423 | AUNIP | -1.0809 | 1.29E-02 |
| ENSG00000137070 | IL11RA | -0.3614 | 1.29E-02 |
| ENSG00000050405 | LIMA1 | 0.5517 | 1.29E-02 |
| ENSG00000177875 | CCDC184 | -0.7955 | 1.29E-02 |
| ENSG00000179104 | TMTC2 | 0.4654 | 1.29E-02 |
| ENSG00000196547 | MAN2A2 | 0.3292 | 1.29E-02 |
| ENSG00000269220 | LINC00528 | 0.4518 | 1.30E-02 |
| ENSG00000163382 | APOA1BP | 0.2894 | 1.31E-02 |
| ENSG00000178814 | OPLAH | 0.9815 | 1.31E-02 |
| ENSG00000151148 | UBE3B | 0.3071 | 1.32E-02 |
| ENSG00000160888 | IER2 | -0.4289 | 1.33E-02 |
| ENSG00000099999 | RNF215 | 0.6614 | 1.33E-02 |
| ENSG00000154537 | FAM27C | 1.1404 | 1.33E-02 |
| ENSG00000273559 | CWC25 | -0.2903 | 1.33E-02 |
| ENSG00000155974 | GRIP1 | 1.0045 | 1.34E-02 |
| ENSG00000198563 | DDX39B | -0.3925 | 1.34E-02 |
| ENSG00000233966 | UBE2SP1 | -0.7137 | 1.34E-02 |
| ENSG00000124942 | AHNAK | 0.3598 | 1.34E-02 |
| ENSG00000179598 | PLD6 | -0.3463 | 1.34E-02 |
| ENSG00000120254 | MTHFD1L | -0.2700 | 1.34E-02 |
| ENSG00000069812 | HES2 | 1.8906 | 1.34E-02 |
| ENSG00000100664 | EIF5 | -0.3768 | 1.34E-02 |
| ENSG00000105676 | ARMC6 | -0.3076 | 1.35E-02 |
| ENSG00000204217 | BMPR2 | 0.3938 | 1.35E-02 |
| ENSG00000217801 | RP11-465B22.3 | 0.7249 | 1.36E-02 |
| ENSG00000185880 | TRIM69 | 0.3033 | 1.38E-02 |
| ENSG00000114646 | CSPG5 | -1.3296 | 1.38E-02 |
| ENSG00000196787 | HIST1H2AG | -0.3417 | 1.38E-02 |
| ENSG00000174444 | RPL4 | -0.3472 | 1.40E-02 |
| ENSG00000163683 | SMIM14 | 0.3604 | 1.40E-02 |
| ENSG00000165409 | TSHR | 0.8768 | 1.42E-02 |
| ENSG00000163517 | HDAC11 | 0.6098 | 1.42E-02 |
| ENSG00000267757 | C19orf83 | 0.4854 | 1.42E-02 |
| ENSG00000269640 | CTD-2521M24.9 | 0.4075 | 1.43E-02 |
| ENSG00000234616 | JRK | -0.3007 | 1.44E-02 |
| ENSG00000138172 | CALHM2 | 0.4915 | 1.44E-02 |
| ENSG00000152104 | PTPN14 | 0.8179 | 1.44E-02 |
| ENSG00000096384 | HSP90AB1 | -0.2761 | 1.45E-02 |
| ENSG00000271122 | RP11-379H18.1 | 0.3765 | 1.45E-02 |
| ENSG00000179152 | TCAIM | 0.3165 | 1.45E-02 |
| ENSG00000204149 | AGAP6 | -0.6450 | 1.45E-02 |
| ENSG00000171793 | CTPS1 | -0.3074 | 1.45E-02 |
| ENSG00000140650 | PMM2 | -0.3346 | 1.45E-02 |
| ENSG00000143554 | SLC27A3 | 0.5667 | 1.45E-02 |
| ENSG00000138380 | CARF | 0.3156 | 1.45E-02 |
| ENSG00000171914 | TLN2 | 0.9073 | 1.45E-02 |
| ENSG00000133466 | C1QTNF6 | 0.7511 | 1.46E-02 |
| ENSG00000272463 | RP11-532F6.3 | 0.4352 | 1.46E-02 |
| ENSG00000126749 | EMG1 | -0.3788 | 1.46E-02 |
| ENSG00000100350 | FOXRED2 | -0.5279 | 1.47E-02 |
| ENSG00000123975 | CKS2 | -0.5004 | 1.47E-02 |
| ENSG00000203865 | ATP1A1-AS1 | 0.6374 | 1.47E-02 |
| ENSG00000204947 | ZNF425 | 0.6921 | 1.47E-02 |
| ENSG00000177666 | PNPLA2 | -0.2917 | 1.47E-02 |
| ENSG00000174175 | SELP | 0.6990 | 1.47E-02 |
| ENSG00000254615 | RP11-395G23.3 | 0.9544 | 1.47E-02 |
| ENSG00000152778 | IFIT5 | 0.6219 | 1.48E-02 |
| ENSG00000167196 | FBXO22 | 0.3199 | 1.50E-02 |
| ENSG00000225439 | BOLA3-AS1 | 0.5863 | 1.50E-02 |
| ENSG00000107937 | GTPBP4 | -0.2985 | 1.50E-02 |
| ENSG00000204381 | LAYN | 0.9228 | 1.50E-02 |
| ENSG00000107719 | PALD1 | 1.5409 | 1.50E-02 |
| ENSG00000183570 | PCBP3 | 0.8129 | 1.54E-02 |
| ENSG00000138448 | ITGAV | 0.3164 | 1.54E-02 |
| ENSG00000084234 | APLP2 | 0.3361 | 1.54E-02 |
| ENSG00000134716 | CYP2J2 | -0.5533 | 1.54E-02 |
| ENSG00000276597 | TRBV11-3 | 0.5188 | 1.54E-02 |
| ENSG00000131669 | NINJ1 | 0.4205 | 1.55E-02 |
| ENSG00000183010 | PYCR1 | -0.3764 | 1.58E-02 |
| ENSG00000136161 | RCBTB2 | 0.5939 | 1.60E-02 |
| ENSG00000054793 | ATP9A | -2.7950 | 1.60E-02 |
| ENSG00000119922 | IFIT2 | 1.3622 | 1.60E-02 |
| ENSG00000133065 | SLC41A1 | 0.3327 | 1.60E-02 |
| ENSG00000042286 | AIFM2 | 0.6007 | 1.61E-02 |
| ENSG00000116815 | CD58 | 0.4231 | 1.65E-02 |
| ENSG00000235251 | RP11-384B12.2 | -0.7211 | 1.65E-02 |
| ENSG00000165169 | DYNLT3 | 0.2790 | 1.65E-02 |
| ENSG00000087088 | BAX | 0.3380 | 1.66E-02 |
| ENSG00000149527 | PLCH2 | 0.6201 | 1.67E-02 |
| ENSG00000130363 | RSPH3 | 0.5138 | 1.67E-02 |
| ENSG00000160219 | GAB3 | 0.4688 | 1.68E-02 |
| ENSG00000159712 | ANKRD18CP | 0.6477 | 1.68E-02 |
| ENSG00000086730 | LAT2 | 0.6449 | 1.69E-02 |
| ENSG00000164105 | SAP30 | 0.3702 | 1.69E-02 |
| ENSG00000162650 | ATXN7L2 | -0.3994 | 1.69E-02 |
| ENSG00000214654 | RP11-27I1.4 | 0.4247 | 1.69E-02 |
| ENSG00000260804 | PKI55 | -0.4027 | 1.70E-02 |
| ENSG00000173548 | SNX33 | 0.5362 | 1.70E-02 |
| ENSG00000134198 | TSPAN2 | 0.9295 | 1.70E-02 |
| ENSG00000110090 | CPT1A | 0.4667 | 1.72E-02 |
| ENSG00000116260 | QSOX1 | 0.2885 | 1.72E-02 |
| ENSG00000176994 | SMCR8 | -0.2730 | 1.73E-02 |
| ENSG00000197603 | C5orf42 | 0.3677 | 1.73E-02 |
| ENSG00000083444 | PLOD1 | 0.4143 | 1.75E-02 |
| ENSG00000143333 | RGS16 | 0.8197 | 1.75E-02 |
| ENSG00000143079 | CTTNBP2NL | 2.2031 | 1.78E-02 |
| ENSG00000117691 | NENF | 0.3098 | 1.79E-02 |
| ENSG00000169083 | AR | -0.5389 | 1.80E-02 |
| ENSG00000167703 | SLC43A2 | 0.7227 | 1.80E-02 |
| ENSG00000090097 | PCBP4 | 0.3328 | 1.80E-02 |
| ENSG00000136816 | TOR1B | 0.2822 | 1.80E-02 |
| ENSG00000213742 | ZNF337-AS1 | 0.5119 | 1.81E-02 |
| ENSG00000153443 | UBALD1 | -0.4972 | 1.83E-02 |
| ENSG00000213261 | EEF1B2P6 | -0.5560 | 1.83E-02 |
| ENSG00000230207 | RPL4P5 | -0.4042 | 1.83E-02 |
| ENSG00000251580 | RP11-539L10.3 | 0.4804 | 1.83E-02 |
| ENSG00000280102 | AL627171.2 | 0.3916 | 1.83E-02 |
| ENSG00000150760 | DOCK1 | -4.4032 | 1.83E-02 |
| ENSG00000115350 | POLE4 | -0.3785 | 1.84E-02 |
| ENSG00000182534 | MXRA7 | 0.4572 | 1.85E-02 |
| ENSG00000013619 | MAMLD1 | 0.9870 | 1.86E-02 |
| ENSG00000148411 | NACC2 | 0.3640 | 1.87E-02 |
| ENSG00000041988 | THAP3 | 0.4137 | 1.87E-02 |
| ENSG00000170846 | AC093323.3 | -0.3022 | 1.87E-02 |
| ENSG00000171490 | RSL1D1 | -0.2802 | 1.87E-02 |
| ENSG00000272452 | N/A | -0.5931 | 1.88E-02 |
| ENSG00000125726 | CD70 | 0.5570 | 1.88E-02 |
| ENSG00000152795 | HNRNPDL | -0.2923 | 1.89E-02 |
| ENSG00000067208 | EVI5 | 0.5877 | 1.90E-02 |
| ENSG00000007312 | CD79B | 0.3106 | 1.95E-02 |
| ENSG00000090376 | IRAK3 | 0.9459 | 1.97E-02 |
| ENSG00000137959 | IFI44L | 0.9713 | 1.99E-02 |
| ENSG00000275764 | RP11-582E3.6 | -0.4909 | 1.99E-02 |
| ENSG00000150687 | PRSS23 | -1.6763 | 2.00E-02 |
| ENSG00000204388 | HSPA1B | -0.4822 | 2.00E-02 |
| ENSG00000090661 | CERS4 | 0.2946 | 2.00E-02 |
| ENSG00000164211 | STARD4 | 0.3682 | 2.03E-02 |
| ENSG00000279943 | FLJ38576 | 0.6916 | 2.04E-02 |
| ENSG00000279407 | AC007191.4 | -0.8729 | 2.04E-02 |
| ENSG00000212747 | FAM127C | 3.1622 | 2.04E-02 |
| ENSG00000078900 | TP73 | -1.4865 | 2.05E-02 |
| ENSG00000246263 | KB-431C1.4 | 0.6890 | 2.05E-02 |
| ENSG00000214182 | PTMAP5 | -0.5556 | 2.05E-02 |
| ENSG00000278535 | DHRS11 | 0.4791 | 2.05E-02 |
| ENSG00000180644 | PRF1 | 0.3488 | 2.05E-02 |
| ENSG00000106211 | HSPB1 | 0.4397 | 2.06E-02 |
| ENSG00000175274 | TP53I11 | 1.5742 | 2.06E-02 |
| ENSG00000065621 | GSTO2 | 0.6406 | 2.06E-02 |
| ENSG00000266088 | RP5-1028K7.2 | 0.7158 | 2.06E-02 |
| ENSG00000101160 | CTSZ | 0.2880 | 2.08E-02 |
| ENSG00000196152 | ZNF79 | 0.3605 | 2.15E-02 |
| ENSG00000080493 | SLC4A4 | 0.4581 | 2.15E-02 |
| ENSG00000146540 | C7orf50 | 0.3180 | 2.21E-02 |
| ENSG00000058668 | ATP2B4 | 0.3442 | 2.21E-02 |
| ENSG00000168395 | ING5 | -0.3513 | 2.21E-02 |
| ENSG00000160703 | NLRX1 | 0.3849 | 2.21E-02 |
| ENSG00000274421 | RP11-386J22.3 | 0.6631 | 2.23E-02 |
| ENSG00000263072 | ZNF213-AS1 | 0.3547 | 2.28E-02 |
| ENSG00000231205 | ZNF826P | 0.4386 | 2.29E-02 |
| ENSG00000111678 | C12orf57 | 0.3736 | 2.30E-02 |
| ENSG00000080546 | SESN1 | 0.4774 | 2.31E-02 |
| ENSG00000103257 | SLC7A5 | -0.2732 | 2.32E-02 |
| ENSG00000105137 | SYDE1 | -4.2671 | 2.35E-02 |
| ENSG00000049192 | ADAMTS6 | 0.5483 | 2.41E-02 |
| ENSG00000197619 | ZNF615 | 0.3001 | 2.41E-02 |
| ENSG00000251136 | RP11-37B2.1 | 0.6788 | 2.41E-02 |
| ENSG00000167992 | VWCE | 0.9289 | 2.42E-02 |
| ENSG00000269051 | CTD-2245F17.3 | 0.6179 | 2.43E-02 |
| ENSG00000135414 | GDF11 | 0.3606 | 2.47E-02 |
| ENSG00000101265 | RASSF2 | 0.3352 | 2.47E-02 |
| ENSG00000234825 | XRCC6P2 | 0.4786 | 2.48E-02 |
| ENSG00000136867 | SLC31A2 | 0.4931 | 2.49E-02 |
| ENSG00000130005 | GAMT | 0.3033 | 2.49E-02 |
| ENSG00000267074 | RP11-1094M14.5 | -0.4276 | 2.50E-02 |
| ENSG00000254667 | AP000783.1 | 0.7512 | 2.50E-02 |
| ENSG00000051108 | HERPUD1 | -0.2990 | 2.50E-02 |
| ENSG00000277476 | RP11-147L13.13 | -0.5738 | 2.50E-02 |
| ENSG00000130244 | FAM98C | 0.3097 | 2.50E-02 |
| ENSG00000104472 | CHRAC1 | -0.2737 | 2.50E-02 |
| ENSG00000138646 | HERC5 | 0.6367 | 2.52E-02 |
| ENSG00000184178 | SCFD2 | -0.3293 | 2.52E-02 |
| ENSG00000074842 | MYDGF | -0.2501 | 2.52E-02 |
| ENSG00000267702 | RP11-53B2.2 | 0.5081 | 2.53E-02 |
| ENSG00000071575 | TRIB2 | -0.4844 | 2.54E-02 |
| ENSG00000177301 | KCNA2 | 0.8311 | 2.54E-02 |
| ENSG00000204256 | BRD2 | -0.3419 | 2.56E-02 |
| ENSG00000106803 | SEC61B | -0.2575 | 2.56E-02 |
| ENSG00000143324 | XPR1 | 0.2950 | 2.56E-02 |
| ENSG00000227398 | KIF9-AS1 | 0.5134 | 2.56E-02 |
| ENSG00000159423 | ALDH4A1 | 0.5793 | 2.58E-02 |
| ENSG00000158887 | MPZ | 0.7283 | 2.61E-02 |
| ENSG00000185189 | NRBP2 | 0.4905 | 2.61E-02 |
| ENSG00000239713 | APOBEC3G | 0.2600 | 2.63E-02 |
| ENSG00000237181 | AC147651.4 | 0.8372 | 2.63E-02 |
| ENSG00000179630 | LACC1 | 0.5738 | 2.63E-02 |
| ENSG00000172687 | ZNF738 | 0.3913 | 2.65E-02 |
| ENSG00000161714 | PLCD3 | 0.8731 | 2.65E-02 |
| ENSG00000168152 | THAP9 | -0.3410 | 2.65E-02 |
| ENSG00000229151 | RP11-348F1.3 | 0.6281 | 2.65E-02 |
| ENSG00000198342 | ZNF442 | 0.5603 | 2.65E-02 |
| ENSG00000196914 | ARHGEF12 | 0.7321 | 2.65E-02 |
| ENSG00000134470 | IL15RA | 0.2995 | 2.70E-02 |
| ENSG00000223865 | HLA-DPB1 | 0.6122 | 2.75E-02 |
| ENSG00000164542 | KIAA0895 | -0.4710 | 2.76E-02 |
| ENSG00000197409 | HIST1H3D | -0.2886 | 2.77E-02 |
| ENSG00000117280 | RAB29 | 0.2449 | 2.78E-02 |
| ENSG00000141378 | PTRH2 | -0.2959 | 2.78E-02 |
| ENSG00000125875 | TBC1D20 | 0.2497 | 2.78E-02 |
| ENSG00000124787 | RPP40 | -0.3570 | 2.82E-02 |
| ENSG00000127528 | KLF2 | -0.3502 | 2.82E-02 |
| ENSG00000114767 | RRP9 | -0.2873 | 2.82E-02 |
| ENSG00000009830 | POMT2 | -0.4763 | 2.83E-02 |
| ENSG00000160191 | PDE9A | 0.6904 | 2.84E-02 |
| ENSG00000136826 | KLF4 | -0.5980 | 2.84E-02 |
| ENSG00000100027 | YPEL1 | 0.3903 | 2.84E-02 |
| ENSG00000151327 | FAM177A1 | 0.2859 | 2.89E-02 |
| ENSG00000134698 | AGO4 | 0.3154 | 2.90E-02 |
| ENSG00000134250 | NOTCH2 | 0.3176 | 2.92E-02 |
| ENSG00000198947 | DMD | -0.8943 | 2.92E-02 |
| ENSG00000278705 | HIST1H4B | -0.3639 | 2.92E-02 |
| ENSG00000105088 | OLFM2 | 0.5089 | 2.92E-02 |
| ENSG00000143891 | GALM | 0.3573 | 2.92E-02 |
| ENSG00000114013 | CD86 | -1.4472 | 2.94E-02 |
| ENSG00000138028 | CGREF1 | -1.1186 | 2.95E-02 |
| ENSG00000278133 | RP11-196G11.5 | -0.3761 | 2.95E-02 |
| ENSG00000256087 | ZNF432 | -0.3062 | 2.96E-02 |
| ENSG00000103175 | WFDC1 | -1.0793 | 2.96E-02 |
| ENSG00000170855 | TRIAP1 | 0.2943 | 2.99E-02 |
| ENSG00000099822 | HCN2 | 0.6523 | 3.01E-02 |
| ENSG00000123384 | LRP1 | 0.7111 | 3.01E-02 |
| ENSG00000162645 | GBP2 | 0.2867 | 3.01E-02 |
| ENSG00000143367 | TUFT1 | 0.5013 | 3.04E-02 |
| ENSG00000268061 | NAPA-AS1 | 0.4794 | 3.04E-02 |
| ENSG00000160190 | SLC37A1 | 0.4240 | 3.06E-02 |
| ENSG00000072310 | SREBF1 | -0.3631 | 3.06E-02 |
| ENSG00000184988 | TMEM106A | 0.4838 | 3.06E-02 |
| ENSG00000161202 | DVL3 | 0.3364 | 3.07E-02 |
| ENSG00000127947 | PTPN12 | 0.3447 | 3.07E-02 |
| ENSG00000123213 | NLN | -0.3175 | 3.11E-02 |
| ENSG00000143061 | IGSF3 | -1.4101 | 3.13E-02 |
| ENSG00000100612 | DHRS7 | 0.2823 | 3.14E-02 |
| ENSG00000196116 | TDRD7 | 0.3045 | 3.15E-02 |
| ENSG00000256235 | SMIM3 | 0.3518 | 3.15E-02 |
| ENSG00000123358 | NR4A1 | -0.7276 | 3.15E-02 |
| ENSG00000168282 | MGAT2 | -0.2686 | 3.15E-02 |
| ENSG00000163528 | CHCHD4 | -0.3577 | 3.15E-02 |
| ENSG00000163564 | PYHIN1 | 0.3346 | 3.15E-02 |
| ENSG00000176531 | PHLDB3 | 0.3475 | 3.17E-02 |
| ENSG00000111674 | ENO2 | 0.3277 | 3.18E-02 |
| ENSG00000167972 | ABCA3 | 0.4118 | 3.26E-02 |
| ENSG00000162433 | AK4 | 0.7133 | 3.27E-02 |
| ENSG00000260336 | N/A | -0.3333 | 3.27E-02 |
| ENSG00000107560 | RAB11FIP2 | -0.2435 | 3.29E-02 |
| ENSG00000048392 | RRM2B | 0.3505 | 3.30E-02 |
| ENSG00000253570 | RNF5P1 | 0.4845 | 3.30E-02 |
| ENSG00000204934 | ATP6V0E2-AS1 | 0.5178 | 3.31E-02 |
| ENSG00000130332 | LSM7 | -0.2942 | 3.31E-02 |
| ENSG00000055332 | EIF2AK2 | 0.5549 | 3.31E-02 |
| ENSG00000158769 | F11R | 0.2773 | 3.32E-02 |
| ENSG00000128272 | ATF4 | -0.2940 | 3.35E-02 |
| ENSG00000196704 | AMZ2 | 0.2867 | 3.38E-02 |
| ENSG00000164904 | ALDH7A1 | 1.3633 | 3.38E-02 |
| ENSG00000215908 | CROCCP2 | -0.4130 | 3.38E-02 |
| ENSG00000255717 | SNHG1 | -0.3548 | 3.38E-02 |
| ENSG00000164134 | NAA15 | -0.2473 | 3.38E-02 |
| ENSG00000037897 | METTL1 | -0.4632 | 3.38E-02 |
| ENSG00000255561 | FDXACB1 | -0.3964 | 3.40E-02 |
| ENSG00000139531 | SUOX | 0.5012 | 3.40E-02 |
| ENSG00000136527 | TRA2B | -0.2556 | 3.40E-02 |
| ENSG00000152454 | ZNF256 | -0.3442 | 3.40E-02 |
| ENSG00000137266 | SLC22A23 | 0.5294 | 3.42E-02 |
| ENSG00000112378 | PERP | 0.3535 | 3.43E-02 |
| ENSG00000168899 | VAMP5 | 0.2915 | 3.44E-02 |
| ENSG00000185917 | SETD4 | -0.3934 | 3.46E-02 |
| ENSG00000175048 | ZDHHC14 | 0.4035 | 3.48E-02 |
| ENSG00000276216 | CH17-373J23.1 | -0.4707 | 3.48E-02 |
| ENSG00000134910 | STT3A | -0.2390 | 3.49E-02 |
| ENSG00000175279 | APITD1 | -0.6189 | 3.49E-02 |
| ENSG00000075213 | SEMA3A | -0.8401 | 3.50E-02 |
| ENSG00000253882 | RP11-61L23.2 | 0.4203 | 3.50E-02 |
| ENSG00000177045 | SIX5 | 0.8770 | 3.51E-02 |
| ENSG00000198125 | MB | 1.9019 | 3.51E-02 |
| ENSG00000181847 | TIGIT | 0.4777 | 3.51E-02 |
| ENSG00000163938 | GNL3 | -0.3221 | 3.54E-02 |
| ENSG00000168060 | NAALADL1 | 0.3673 | 3.54E-02 |
| ENSG00000013441 | CLK1 | -0.3015 | 3.56E-02 |
| ENSG00000067533 | RRP15 | -0.2775 | 3.56E-02 |
| ENSG00000124225 | PMEPA1 | 0.4532 | 3.65E-02 |
| ENSG00000261474 | RP11-452L6.1 | -0.6242 | 3.66E-02 |
| ENSG00000213160 | KLHL23 | 0.4156 | 3.66E-02 |
| ENSG00000166598 | HSP90B1 | -0.2470 | 3.67E-02 |
| ENSG00000156711 | MAPK13 | 0.2686 | 3.68E-02 |
| ENSG00000253182 | RP11-486M23.2 | -4.1322 | 3.69E-02 |
| ENSG00000241316 | SUCLG2-AS1 | 0.4508 | 3.70E-02 |
| ENSG00000266946 | MRPL37P1 | 0.5819 | 3.71E-02 |
| ENSG00000081913 | PHLPP1 | 0.3940 | 3.71E-02 |
| ENSG00000197961 | ZNF121 | -0.3012 | 3.71E-02 |
| ENSG00000119673 | ACOT2 | -0.3674 | 3.71E-02 |
| ENSG00000164304 | CAGE1 | -0.5336 | 3.71E-02 |
| ENSG00000270964 | RP11-502I4.3 | 0.6226 | 3.72E-02 |
| ENSG00000135148 | TRAFD1 | 0.3326 | 3.73E-02 |
| ENSG00000234882 | EIF3EP1 | 0.6746 | 3.74E-02 |
| ENSG00000170017 | ALCAM | 0.3181 | 3.75E-02 |
| ENSG00000183628 | DGCR6 | 0.4853 | 3.75E-02 |
| ENSG00000136271 | DDX56 | -0.2435 | 3.77E-02 |
| ENSG00000246526 | RP11-539L10.2 | -0.6582 | 3.77E-02 |
| ENSG00000254531 | FLJ20021 | 0.6212 | 3.77E-02 |
| ENSG00000139998 | RAB15 | 0.5159 | 3.90E-02 |
| ENSG00000217555 | CKLF | 0.3614 | 3.93E-02 |
| ENSG00000154845 | PPP4R1 | 0.2589 | 3.93E-02 |
| ENSG00000106404 | CLDN15 | -0.6304 | 3.94E-02 |
| ENSG00000258486 | RN7SL1 | 0.2704 | 3.94E-02 |
| ENSG00000137434 | C6orf52 | 0.8335 | 3.96E-02 |
| ENSG00000103056 | SMPD3 | 0.8610 | 3.97E-02 |
| ENSG00000272602 | ZNF595 | 0.4902 | 3.97E-02 |
| ENSG00000260267 | RP11-452L6.5 | -0.2825 | 3.97E-02 |
| ENSG00000135046 | ANXA1 | -0.3748 | 4.01E-02 |
| ENSG00000135378 | PRRG4 | 1.0340 | 4.01E-02 |
| ENSG00000137968 | SLC44A5 | 0.3608 | 4.01E-02 |
| ENSG00000159335 | PTMS | 0.7043 | 4.01E-02 |
| ENSG00000198026 | ZNF335 | -0.2863 | 4.01E-02 |
| ENSG00000058600 | POLR3E | -0.2366 | 4.08E-02 |
| ENSG00000124523 | SIRT5 | 0.3280 | 4.11E-02 |
| ENSG00000261428 | RP11-16P6.1 | -0.6061 | 4.12E-02 |
| ENSG00000260101 | RP11-568N6.1 | -0.5848 | 4.12E-02 |
| ENSG00000011028 | MRC2 | 0.5901 | 4.12E-02 |
| ENSG00000187514 | PTMA | -0.2339 | 4.13E-02 |
| ENSG00000012061 | ERCC1 | -0.2747 | 4.13E-02 |
| ENSG00000278828 | HIST1H3H | -0.2655 | 4.13E-02 |
| ENSG00000166349 | RAG1 | 0.7959 | 4.13E-02 |
| ENSG00000029993 | HMGB3 | 0.3855 | 4.14E-02 |
| ENSG00000197153 | HIST1H3J | -0.4166 | 4.14E-02 |
| ENSG00000065717 | TLE2 | 0.6681 | 4.14E-02 |
| ENSG00000214113 | LYRM4 | -0.2795 | 4.14E-02 |
| ENSG00000265185 | SNORD3B-1 | -0.5803 | 4.14E-02 |
| ENSG00000179388 | EGR3 | -0.6572 | 4.14E-02 |
| ENSG00000006652 | IFRD1 | -0.2983 | 4.17E-02 |
| ENSG00000171777 | RASGRP4 | 0.9813 | 4.18E-02 |
| ENSG00000124575 | HIST1H1D | -0.3317 | 4.19E-02 |
| ENSG00000272686 | RP11-390E23.6 | 0.4687 | 4.19E-02 |
| ENSG00000237036 | ZEB1-AS1 | 0.3834 | 4.19E-02 |
| ENSG00000106086 | PLEKHA8 | 0.3943 | 4.19E-02 |
| ENSG00000204519 | ZNF551 | -0.2651 | 4.21E-02 |
| ENSG00000140104 | C14orf79 | -0.6108 | 4.21E-02 |
| ENSG00000138758 | SEPT11 | 0.4634 | 4.23E-02 |
| ENSG00000104835 | SARS2 | -0.4784 | 4.23E-02 |
| ENSG00000099377 | HSD3B7 | 0.7905 | 4.25E-02 |
| ENSG00000196867 | ZFP28 | -0.3283 | 4.28E-02 |
| ENSG00000260253 | RP4-676L2.1 | -1.5637 | 4.29E-02 |
| ENSG00000164512 | ANKRD55 | -0.3900 | 4.30E-02 |
| ENSG00000163596 | ICA1L | 0.5597 | 4.30E-02 |
| ENSG00000235944 | ZNF815P | -0.3818 | 4.30E-02 |
| ENSG00000227028 | SLC8A1-AS1 | 0.6731 | 4.30E-02 |
| ENSG00000166503 | HDGFRP3 | -0.3527 | 4.30E-02 |
| ENSG00000131089 | ARHGEF9 | 0.2748 | 4.30E-02 |
| ENSG00000226054 | MEMO1P1 | -0.7147 | 4.30E-02 |
| ENSG00000054392 | HHAT | 0.5507 | 4.30E-02 |
| ENSG00000198756 | COLGALT2 | -1.3336 | 4.30E-02 |
| ENSG00000273004 | GS1-279B7.2 | -0.5759 | 4.30E-02 |
| ENSG00000182557 | SPNS3 | 0.4299 | 4.32E-02 |
| ENSG00000261236 | BOP1 | -0.2551 | 4.32E-02 |
| ENSG00000271870 | RP11-97C16.1 | 0.6515 | 4.34E-02 |
| ENSG00000069399 | BCL3 | 0.6008 | 4.34E-02 |
| ENSG00000245532 | NEAT1 | 0.4950 | 4.38E-02 |
| ENSG00000152229 | PSTPIP2 | 0.4746 | 4.38E-02 |
| ENSG00000184675 | AMER1 | -0.2988 | 4.39E-02 |
| ENSG00000160818 | GPATCH4 | -0.3172 | 4.39E-02 |
| ENSG00000116191 | RALGPS2 | 0.3694 | 4.43E-02 |
| ENSG00000151773 | CCDC122 | 0.5477 | 4.43E-02 |
| ENSG00000135740 | SLC9A5 | 0.7581 | 4.43E-02 |
| ENSG00000142920 | AZIN2 | 0.4494 | 4.43E-02 |
| ENSG00000109189 | USP46 | -0.4421 | 4.43E-02 |
| ENSG00000250889 | LINC01336 | -0.5410 | 4.43E-02 |
| ENSG00000114745 | GORASP1 | 0.2828 | 4.44E-02 |
| ENSG00000147403 | RPL10 | -0.2908 | 4.44E-02 |
| ENSG00000136770 | DNAJC1 | 0.2455 | 4.46E-02 |
| ENSG00000278879 | AP000560.3 | 0.8081 | 4.48E-02 |
| ENSG00000066027 | PPP2R5A | 0.2608 | 4.50E-02 |
| ENSG00000090554 | FLT3LG | -0.5729 | 4.50E-02 |
| ENSG00000174327 | SLC16A13 | 0.5946 | 4.52E-02 |
| ENSG00000138686 | BBS7 | 0.3245 | 4.53E-02 |
| ENSG00000141013 | GAS8 | 0.5426 | 4.53E-02 |
| ENSG00000275454 | CTD-2026K11.4 | -0.4715 | 4.53E-02 |
| ENSG00000177469 | PTRF | 1.1401 | 4.54E-02 |
| ENSG00000278376 | RP11-158I9.8 | 0.6436 | 4.54E-02 |
| ENSG00000172375 | C2CD2L | -0.2564 | 4.56E-02 |
| ENSG00000167721 | TSR1 | -0.2422 | 4.62E-02 |
| ENSG00000159884 | CCDC107 | 0.2742 | 4.62E-02 |
| ENSG00000132334 | PTPRE | 0.2602 | 4.62E-02 |
| ENSG00000134291 | TMEM106C | 0.2540 | 4.62E-02 |
| ENSG00000096060 | FKBP5 | 0.3171 | 4.62E-02 |
| ENSG00000245648 | RP11-277P12.20 | 2.2249 | 4.62E-02 |
| ENSG00000232160 | RAP2C-AS1 | 0.4364 | 4.65E-02 |
| ENSG00000136193 | SCRN1 | 0.5492 | 4.65E-02 |
| ENSG00000162711 | NLRP3 | 0.6169 | 4.65E-02 |
| ENSG00000138835 | RGS3 | 0.3599 | 4.65E-02 |
| ENSG00000116016 | EPAS1 | 1.0545 | 4.65E-02 |
| ENSG00000092199 | HNRNPC | 0.2102 | 4.67E-02 |
| ENSG00000188313 | PLSCR1 | 0.5799 | 4.67E-02 |
| ENSG00000184371 | CSF1 | 0.4412 | 4.67E-02 |
| ENSG00000240370 | U47924.6 | 0.4925 | 4.69E-02 |
| ENSG00000204392 | LSM2 | 0.2417 | 4.69E-02 |
| ENSG00000075790 | BCAP29 | 0.3008 | 4.69E-02 |
| ENSG00000029363 | BCLAF1 | -0.2445 | 4.69E-02 |
| ENSG00000070718 | AP3M2 | -0.3449 | 4.69E-02 |
| ENSG00000165271 | NOL6 | -0.2840 | 4.69E-02 |
| ENSG00000266208 | CTD-2267D19.3 | 0.4052 | 4.70E-02 |
| ENSG00000125170 | DOK4 | 0.4466 | 4.70E-02 |
| ENSG00000109321 | AREG | -0.5253 | 4.70E-02 |
| ENSG00000270629 | NBPF14 | 0.3880 | 4.70E-02 |
| ENSG00000138271 | GPR87 | 2.4780 | 4.71E-02 |
| ENSG00000157680 | DGKI | -2.0404 | 4.71E-02 |
| ENSG00000184678 | HIST2H2BE | -0.3568 | 4.73E-02 |
| ENSG00000012232 | EXTL3 | 0.3009 | 4.73E-02 |
| ENSG00000171408 | PDE7B | 0.7108 | 4.73E-02 |
| ENSG00000115884 | SDC1 | -2.9435 | 4.73E-02 |
| ENSG00000197279 | ZNF165 | -0.4460 | 4.73E-02 |
| ENSG00000159860 | TCAF2P1 | 0.4504 | 4.73E-02 |
| ENSG00000152689 | RASGRP3 | 0.3946 | 4.73E-02 |
| ENSG00000124383 | MPHOSPH10 | -0.3674 | 4.77E-02 |
| ENSG00000258875 | CTD-2547L24.3 | 0.3314 | 4.77E-02 |
| ENSG00000278784 | RP11-468E2.11 | -0.4984 | 4.77E-02 |
| ENSG00000013583 | HEBP1 | 0.5008 | 4.77E-02 |
| ENSG00000173960 | UBXN2A | 0.2486 | 4.77E-02 |
| ENSG00000172296 | SPTLC3 | 0.6915 | 4.77E-02 |
| ENSG00000138604 | GLCE | 0.3213 | 4.81E-02 |
| ENSG00000088833 | NSFL1C | 0.2304 | 4.86E-02 |
| ENSG00000089127 | OAS1 | 0.6344 | 4.88E-02 |
| ENSG00000104549 | SQLE | -0.3468 | 4.88E-02 |
| ENSG00000012822 | CALCOCO1 | 0.2414 | 4.92E-02 |
| ENSG00000107438 | PDLIM1 | 0.6696 | 4.92E-02 |
| ENSG00000128590 | DNAJB9 | -0.2741 | 4.99E-02 |
| ENSG00000085788 | DDHD2 | 0.2496 | 4.99E-02 |
| ENSG00000249855 | EEF1A1P19 | -0.5525 | 4.99E-02 |
