# Supplementary material for: Transcriptomic Analysis Implicates the p53 Signaling Pathway in the Establishment of HIV-1 Latency in Central Memory CD4 T Cells in an In Vitro Model
Source: PLoS Pathog. 2016 Nov 29;12(11):e1006026. doi: 10.1371/journal.ppat.1006026 (PMC5127598; doi:10.1371/journal.ppat.1006026)
Supplement: S2 Table — This table demonstrates genes which overlap in significance and direction between the TCM model [20] presented in the current study and the model of Iglesias-Ussel et al. [9]. Asterisks indicate involvement with p53 signaling. (DOCX) [file ppat.1006026.s005.docx]

| Upregulated | | | | Downregulated | |
| --- | --- | --- | --- | --- | --- |
|  | | | |  | |
| *ABHD4* | *DDB2** | *KIF9-AS1* | *RGL1* | *ATF4* | *NAA15* |
| *ACTA2** | *DNAJC1* | *MAN2A2* | *RNFT1* | *BOP1* | *NOL6* |
| *AEN* | *DRAM1** | *MICAL1* | *RPL39L* | *C10ORF2* | *NOP16* |
| *ALDH4A1* | *FDXR** | *MPZL1* | *RTN4RL1* | *C1QBP* | *PDIA5* |
| *APOBEC3H* | *FOXP4* | *MSRB2* | *SAMD4A* | *C2CD2L* | *PIGW* |
| *ASTN2* | *GADD45A** | *NBPF14* | *SIDT2* | *CROCCP2* | *POGLUT1* |
| *ATF7IP2* | *GAMT* | *NEAT1* | *SLC22A23* | *GNL3* | *PTMA* |
| *AZIN2* | *GBP2* | *NR1H3* | *SMIM3* | *HDGFRP3* | *RORC* |
| *BAX* | *GNA15* | *NRBP2* | *SNTA1* | *HNRNPDL* | *RSL1D1* |
| *BBC3** | *GSS* | *PCBP4* | *SPATA18* | *HSP90AB1* | *SFPQ* |
| *C11orf24* | *GSTM3* | *PDE7B* | *SYTL3* | *IFRD2* | *STT3A* |
| *C12orf5* | *GZMA* | *PHLDA3* | *TM7SF3* | *ING5* | *TRIB2* |
| *C7orf50* | *IDNK* | *PHPT1* | *TNFRSF10B** | *KLF2* | *TSR1* |
| *CACNB3* | *IFI44* | *PMEPA1* | *TRIAP1* | *MEPCE* | *TWISTNB* |
| *CD70* | *IFIT2* | *PSMG3-AS1* | *TRIB1* | *METTL1* | *UBE2S* |
| *CELSR2* | *IL18RAP* | *RAB15* | *VWCE* | *MIDN* | *USP46* |
| *CKLF* |  |  |  | *MYC* | *ZNF335* |
