# Supplementary material for: Transcriptomic Analysis Implicates the p53 Signaling Pathway in the Establishment of HIV-1 Latency in Central Memory CD4 T Cells in an In Vitro Model
Source: PLoS Pathog. 2016 Nov 29;12(11):e1006026. doi: 10.1371/journal.ppat.1006026 (PMC5127598; doi:10.1371/journal.ppat.1006026)
Supplement: S1 Supplemental Methods — This file contains all supplemental methods for total RNA-Seq data analysis including; quantification of spliced transcripts, gene differential expression analysis, biological scaling normalization, RUVSeq normalization, as well as supplemental methods for RT-qPCR validation procedures. (PDF) [file ppat.1006026.s006.pdf]

SUPPLEMENTAL METHODS

1.1. Total RNA-Seq Data Analysis

FASTQ files from the HiSeq2000 instrument were provided by Expression Analysis Inc. (Q^2 Solutions, NC, USA) and assessed for quality using the Fastx-Toolkit suite available from the Hannon lab (http://hannonlab.cshl.edu/fastx_toolkit/). Reads were then mapped to the hg38 (human genome released on December 24th) 2013 using Tophat (version 2.0.13) [1]. Default settings were used with options -bowtie1 and --no-coverage-search. Mapped reads were sorted by name and converted to sam files using samtools [2]. Sam files were counted against the GENCODE [3] gene annotation (version 21) with HTSeq 0.6.1p2 and htseq-count [4] using the default settings. All level 3 annotations were removed from the GENDCODE transcript file prior to counting as those members are automatically annotated and not manually verified. Any gene which did not achieve at least 1 count per million reads (cpm) in at least 4 (25%) samples (either resting or activated) was removed.

Total reads were separately mapped to the HIV genome reference (Genebank accession number AF324493) with Tophat utilizing the following alterations from default settings: max segment intron length of 10000, max coverage intron length of 10000, 0 segment mismatches, 0 mismatches, and no coverage search. These options were selected to reduce the possibility of a read being erroneously assigned to the HIV genome due to possible sequence similarity to hg38. Reads were counted against the HIV genome using HTSeq as described previously.

Finally, all reads were mapped to the 92 ERCCs (reference available from ThermoFisher Scientific, https://tools.thermofisher.com/content/sfs/manuals/cms_095047.txt) using bowtie with 0 mismatches and the --best option. Any ERCC that did not achieve greater than 5 reads in at least 4 samples was removed leaving 67 ERCCs. Reads that mapped to the ERCC reference were then re-mapped to hg38 and HIV to confirm that these reads mapped solely to ERCCs and were not cross mapping to any other reference (data not shown).

1.2. Quantification of Spliced Transcripts

To determine HIV read splicing, a method developed by [5] was utilized. This method counts reads that pass through two HIV splice junctions, D1 (directly after the LTR region), and D4 (splice junction between tat-rev and vpu) that define multiply spliced (MS), singly spliced (SS), and unspliced (US) transcripts. Reads passing through the junction D1 belong to US transcripts. Reads which align to the left of this junction, but are broken at D1 and align to other sections of HIV, correspond to either SS or MS transcripts (SS+MS). Reads overlapping the D4 junction correspond to reads from either US or SS transcripts (US+SS). Finally, reads broken at the D4 junction correspond to reads from MS transcripts. The percentage of reads from US transcripts is estimated by obtaining the fraction of reads that pass through the D1 junction. The percentage of reads from MS transcripts is estimated by obtaining the fraction of reads that break at the D4 junction. The percentage of reads from SS transcripts is estimated by subtracting the estimated percentages of US and MS reads from 100%.

1.3. Biological Scaling Normalization (BSN) From Resting to Activated States.

The large difference in cellular RNA content between the activated versus resting states resulted from transcriptional amplification. Therefore, Biological Scaling Normalization (BSN) described in [6] was utilized to adjust read levels based upon internal ERCC spike-in controls. We chose 11 ERCCs (ERCC_00130, ERCC_00002, ERCC_00096, ERCC_00074, ERCC_00004, ERCC_00113, ERCC_00046, ERCC_00171, ERCC_00136, ERCC_00003, and ERCC_00108) with read counts above 1000 across all samples for this analysis as low abundance ERCCs were not readily detected in activated samples (data not shown). For each ERCC, an average was calculated across the resting state only. This average was divided by each ERCC count in individual donors resulting in an ERCC scale factor based upon the resting state for each sample. These scale factors were averaged across all 11 ERCCs to obtain a sample specific scaling factor. These scaling factors were then used in the BSN method. Briefly, the raw counts in each state were summed together to generate a raw library size for each sample. This library size was averaged to generate a common library size and then converted to a pseudo library size for each donor by multiplication with the appropriate scaling factors (Equation 1). Then, for each gene and sample, the read concentration for a given transcript was calculated (Equation 2).

$$X_{ij} = CommonLibrarySize * ScaleFactor \quad (1)$$

$$C_{ij} = \frac{R_{ij}}{R_j} \quad (2)$$

Where C_{ij} is the concentration of transcript *i* relative to sample *j*, R_{ij} is the read count of gene *i* in sample *j*, and R_j is the total number of reads in sample *j*. The final step is to assign the previously calculated pseudo reads to genes based upon their concentration to obtain a scaled normalized dataset (Equation 3).

$$P_{ij} = C_{ij} * X_{ij} \quad (3)$$

1.4. RUVSeq Normalization Within the Resting State

Transcriptional amplification did not occur when comparing latently infected to uninfected samples since both conditions were in the resting state. Therefore RUVSeq [7] was used for data normalization in order to remove unwanted variation across samples. This method was appropriate within the resting dataset because it removes variation based upon sample differences not attributable to differing conditions, but rather due to technical variation in sample prep and processing. RUVSeq adjusts for nuisance technical effects by performing factor analysis on the ERCC spike in controls. This program creates a generalized linear model where the read counts are regressed onto known covariates of interest (e.g. the covariate of treatment effect) and factors of unwanted variation of unknown origin. RUVSeq then calculates a vector of unwanted variation based upon the expression of the negative controls (ERCCs) that may be used in linear modeling approaches to differential gene expression where the unwanted variation is assigned to a variable that may then be removed from the comparison of states (uninfected vs. latently infected in our case). This vector is then used as a blocking variable during differential expression. ERCC results that passed filtering (67 ERCCs) were combined

with host gene mapping results and normalized using upper quartile normalization available in RUVSeq.

1.5. Differential Expression in the Resting State

Following read count normalization, differential expression was performed with EdgeR [8]. This program utilizes an overdispersed Poisson model which accounts for both biological and technical variability coupled with an empirical Bayes method to moderate the degree of overdispersion. Afterwards the data was fit to a generalized linear model (GLM) and a GLM likelihood ratio test was performed to determine if the coefficient representing the contrast between the conditions of interest was equal to 0 indicating no differential expression. The donor number was used as a blocking variable due the paired nature of the samples. Additionally, the vector of unwanted variation provided by RUVSeq was also used as a blocking variable. Significance for differential expression was set at a FDR corrected p-value < 0.05.

1.6. RT-qPCR Validation

Total RNA was reverse transcribed using the High Capacity cDNA Reverse Transcription Kit (Life Technologies) following manufacturer's instructions. RT-qPCR was performed for each target using TaqMan Universal PCR Master Mix (No AmpErase UNG) on the ABI 7900HT Fast Real-Time PCR System (Applied Biosystems, CA, USA). Thermal cycling conditions were 95˚C for 10 minutes followed by 40 cycles of 95˚C for 15 seconds and 60˚C for 1 minute. Changes in gene expression were calculated using the $2^{-\Delta\Delta CT}$ method with the spike-in ERCC control ERCC_00130, as the normalizer. Targets validated by RT-qPCR were *GADD45A, MDM2, FAS, TNFRSF10B, TP53I3, BBC, IL2, KLF2*, and *HEXIM1* (Taqman Assay IDs Hs00169255_m1, Hs01066930_m1, Hs00163653_m1, Hs00366278_m1, Hs00936520_m1, Hs00248075_m1, Hs00174114_m1, Hs00360439_g1, Hs00538918_s1, respectively). In addition, levels of unspliced *Gag*, multiply spliced *Tat-Rev* and poly-adenylated HIV transcripts were measured using custom TaqMan Gene Expression Assays using primer and probe sequences described previously [9] (http://www.bio-protocol.org/e1492).

REFERENCES

1. Trapnell C, Pachter L, Salzberg SL (2009) TopHat: discovering splice junctions with RNA-Seq. Bioinformatics 25: 1105-1111.
2. Li H, Handsaker B, Wysoker A, Fennell T, Ruan J, et al. (2009) The Sequence Alignment/Map format and SAMtools. Bioinformatics 25: 2078-2079.
3. Harrow J, Frankish A, Gonzalez JM, Tapanari E, Diekhans M, et al. (2012) GENCODE: the reference human genome annotation for The ENCODE Project. Genome Res 22: 1760-1774.
4. Anders S, Pyl PT, Huber W (2015) HTSeq--a Python framework to work with high-throughput sequencing data. Bioinformatics 31: 166-169.
5. Mohammadi P, di Iulio J, Munoz M, Martinez R, Bartha I, et al. (2014) Dynamics of HIV latency and reactivation in a primary CD4+ T cell model. PLoS Pathog 10: e1004156.
6. Aanes H, Winata C, Moen LF, Ostrup O, Mathavan S, et al. (2014) Normalization of RNA-sequencing data from samples with varying mRNA levels. PLoS One 9: e89158.

7. Risso D, Ngai J, Speed TP, Dudoit S (2014) Normalization of RNA-seq data using factor analysis of control genes or samples. Nat Biotechnol 32: 896-902.
8. Robinson MD, McCarthy DJ, Smyth GK (2010) edgeR: a Bioconductor package for differential expression analysis of digital gene expression data. Bioinformatics 26: 139-140.
9. Massanella M, Gianella, S., Lada, S. M., Richman, D. D. and Strain, M. C. (2015) Quantification of Total and 2-LTR (Long terminal repeat) HIV DNA, HIV RNA and Herpesvirus DNA in PBMCs. Bio-protocol 5: e1492.
